# Impact of Resistance and Endurance Training on Ghrelin and Plasma Leptin Levels in Overweight and Obese Subjects

**DOI:** 10.3390/ijms25158067

**Published:** 2024-07-24

**Authors:** Brindusa Ilinca Mitoiu, Roxana Nartea, Roxana Steliana Miclaus

**Affiliations:** 1 Clinical Department 9, Carol Davila University of Medicine and Pharmacy, 050474 Bucharest, Romania; brindusa.mitoiu@umfcd.ro; 2 Prof. Dr. Agrippa Ionescu Clinical Emergency Hospital, 077016 Bucharest, Romania; 3 National Institute for Rehabilitation, Physical Medicine and Balneoclimatology, 030079 Bucharest, Romania; 4 Department of Fundamental, Preventive, and Clinical Disciplines, Faculty of Medicine, Transilvania University of Brasov, 500036 Brasov, Romania; roxicum@unitbv.ro; 5 Neurorehabilitation Department, Clinical Hospital of Psychiatry and Neurology, 500036 Brasov, Romania

**Keywords:** ghrelin, leptin, appetite-related peptides, exercise, training, physical activity, overweight, obesity

## Abstract

Exercise training is a valuable tool for improving body weight and composition in overweight or obese adults, which leads to a negative energy balance. It is relevant to consider whether exercise can help people lose weight or prevent weight gain because any energy expended in exercise increases the severity of hunger and promotes food consumption. Over the past decade, the identification of the circulating peptide ghrelin, which alerts the brain to the body’s nutritional state, has significantly expanded our understanding of this homeostatic mechanism that controls appetite and body weight. To shed more light on this issue, we decided to investigate the effects of resistance and endurance training on plasma ghrelin and leptin levels. In addition, we sought to understand the mechanisms by which acute and chronic exercise can regulate hunger. This review analyzes studies published in the last fifteen years that focused on changes suffered by ghrelin, leptin, or both after physical exercise in overweight or obese individuals. Most studies have shown a decrease in leptin levels and an increase in ghrelin levels in these cases. Exercise regimens that support weight maintenance need further investigation.

## 1. Introduction

An imbalance between energy intake and expenditure leads to obesity when energy intake through food consumption exceeds energy expenditure. Three factors make up energy expenditure: physical activity, postprandial thermogenesis, and basal metabolism. The latter factor is believed to be the most important and has been shown to be reduced in obese individuals, whereas food consumption usually increases.

Dietary practices are associated with metabolic health and have been demonstrated to be predictive of chronic non-communicable diseases such as type 2 diabetes mellitus, coronary heart disease, stroke, and certain types of cancer [1,2]. Many environmental, social, psychological, and physiological factors impact food-related behaviors, creating an intricate and often unpredictable interaction that influences food consumption. This complexity adds a layer of intrigue to the challenge of controlling dietary practices and regulating food intake. From a physiological point of view, the hormonal balance that indicates both satisfaction and hunger significantly controls the desire to eat. 

Food intake involves hormonal communication from the gastrointestinal tract, adipocytes, and other parts of the body (i.e., fat or adipose cells). The hypothalamus generally has primary control over food intake. Understanding and addressing these factors are crucial in controlling dietary practices, especially in obese and overweight individuals.

Leptin and ghrelin are two key players in this complex system. Ghrelin, a well-known gut hormone, increases the urge and sensation of hunger. Conversely, the body produces a variety of signals that decrease appetite, including pancreatic hormones like insulin and pancreatic polypeptide (PP) and gut hormones like leptin. Mainly, endothelial cells in the pancreas and digestive system release these hormones postprandially. Increased levels of these hormones set off a series of events that limit food intake, suppress food-seeking behaviors, and produce a false sense of fullness [3,4,5].

Apart from their reactions during feeding and fasting, these hormones that control hunger also exhibit variations during and after physical activity. 

Physical exercise is undoubtedly beneficial for health. The processes that mediate these advantages are still not entirely known, though. Interest has been expressed in the relationship between exercise and ghrelin or leptin release since both hormones affect energy homeostasis, body composition, and glucose and lipid metabolism.

Several studies have investigated the effects of both acute and long-term exercise on hormones linked to hunger production individually. The findings are conflicting, with circulating ghrelin and leptin levels shown to rise, fall, or remain unchanged in response to acute or long-term physical activity. These contradictory data spark curiosity and encourage further research to uncover the truth.

In the last fifteen years, the topic has been the focus of additional research, and new information has become accessible. We conducted a thorough literature analysis in this regard to obtain an updated perspective on how resistance and endurance training affect the production of ghrelin and leptin levels in obese and overweight individuals. Our main goal is to explore the effect of exercise on ghrelin and leptin levels according to the research subject, activity level, and exercise timing. Additionally, we will try to underline mechanisms via which acute and chronic exercise can regulate hunger.

### 1.1. Ghrelin 

#### 1.1.1. General Information

Ghrelin is a 28-amino-acid peptide discovered by Kojima et al. in 1999 [6]; it functions as a natural ligand of the orphan growth hormone secretagogue receptor type 1a (GHS-R 1a) in gastric extracts. Although the stomach is primarily associated with ghrelin production, the ghrelin gene is widely dispersed throughout the body, among other tissues such as the intestines, kidneys, pancreas, placenta, testicles, pituitary gland, and brain. Ghrelin has two primary chemical forms: des-acyl ghrelin and acylated ghrelin. The former fulfills most of ghrelin’s activities, making up less than 10% of the total circulating ghrelin [7]. 

It has been demonstrated that ghrelin is essential for regulating the release of growth hormone [8,9]. Still, it is also involved in several other physiological processes, including food intake, behavior linked to rewards, glucose homeostasis (inhibiting insulin secretion and regulating the synthesis and metabolism of glucose and glycogen), motility of the gastrointestinal system, prevention of muscle atrophy (by supporting the differentiation and fusion of muscle fibers), reducing the activity of the sympathetic nervous system (for example, it improves the prognosis regarding survival in the case of myocardial infarction), a role in regulating bone growth through the differentiation and proliferation of osteoblasts (high levels of this hormone are recorded in metastatic cancer cases), and so on [10,11,12]. 

Furthermore, biological materials can also include des-acyl ghrelin, a non-acylated version of ghrelin. In contrast to ghrelin, des-acyl ghrelin does not bind to GHSR at physiological levels, and its physiological function is still poorly understood. 

The literature now uses several names to refer to ghrelin and des-acyl ghrelin. In 2023, the use of GHSR for the receptor and LEAP2 for liver-expressed antimicrobial peptide 2 (a recently recognized endogenous GHSR antagonist/inverse agonist) [13] was suggested.

#### 1.1.2. Obesity and Ghrelin

Ghrelin is involved in multiple physiological functions, such as regulating growth hormone (GH) secretion [13,14,15], adiposity [16,17,18], gastric acid secretion [10,17], and gut motility [14,16,19]. Ghrelin is a crucial player in anorexic behaviors related to appetite stimulation and body weight management [20]. Ghrelin expression in the stomach rises during fasting and decreases within 1 h of having a meal [21]. Postprandially, the decrease in plasma ghrelin levels is also proportional to the amount of calories consumed, underlining ghrelin’s role as a hunger signal [11,12,16]. Ghrelin levels and hunger scores are correlated [10]. Ghrelin is also associated with the inner digestive contractions of the stomach in rats, as well as with the stimulation of gastric acid secretion and motility [17,22].

Age, lactation, and sex hormones can also influence ghrelin secretion and mRNA expression of the octanoylating enzyme ghrelin O-acyltransferase (GOAT), critically affecting ghrelin activity [21]. Studies have shown that ghrelin secretion is upregulated in patients with anorexia and cachexia, while it is downregulated in patients with hyperphagia and obesity [20,23,24]. Moreover, studies have recently reported that decreased basal and postprandial ghrelin levels were observed in age-matched female patients with functional dyspepsia (FD) compared to healthy controls [20,23]. Suppressed ghrelin responses were significantly correlated with meal-related symptoms in female patients with FD experiencing higher bloating and satiety ratings [25]. Their findings also revealed abnormal ghrelin responses in functional GI disorders. Therefore, how ghrelin is controlled in such situations needs to be clarified.

Fasting plasma levels of ghrelin in obese subjects are significantly lower than those in non-obese subjects [12,26,27]. Plasma-acylated ghrelin levels are significantly and negatively correlated with the basal metabolic index; visceral, subcutaneous, and total fat area; and serum insulin levels in type 2 diabetic patients. In these patients, plasma acylated ghrelin levels were significantly associated with serum insulin levels after adjustments for body mass index (BMI) [26,28,29]. Plasma ghrelin concentrations increase after weight loss, and the increase in ghrelin levels is positively correlated with the extent of weight loss [24,26,27,29,30]. Ghrelin may play a role in the adaptive response that limits the amount of weight that may be lost by dieting [13,28,31,32,33,34]. 

### 1.2. Leptin

#### 1.2.1. General Information

Leptin is a 167-amino-acid hormone discovered by Douglas Coleman and Jeffrey Friedman in 1994, first known as the obesity hormone; it is synthesized predominantly (but not entirely) by white adipose tissue [35]. The leptin gene (*ob*) is situated on chromosome 7q31.3 [36]. Leptin functions by attaching itself to leptin receptors (LRs) on the cell surface of cardiac, perivascular intestinal, hepatic, and neural tissues [37].

The variables that most influence the levels of circulating plasma leptin are gender, metabolic hormones, and total body mass index (BMI). The amount of leptin is proportional to the quantity of body fat mass. Compared to men, women’s levels of circulating leptin are higher [37,38,39]. 

There are at least six isoforms of leptin receptors (LepRa, LepRb, LepRc, LepRd, LepRe, and LepRf), each of which has a unique intracellular domain and a common leptin-binding domain. Lep-Rb (long isoform) is the primary source of leptin-specific effects, since it is the only isoform with the intracellular motif required for leptin-mediated activation of the Janus kinase/signal transducers and activators of transcription (JAK-STAT) signaling pathway in the hypothalamus. LepRb is a regular class I cytokine receptor that does not have internal kinase activity. Leptin’s binding to LepRb facilitates the activation of Janus kinase 2 (JAK2), followed by autophosphorylation and the phosphorylation of three tyrosine residues in LepRb (Y985, Y1077, and Y1138). These phosphorylated residues interact with signaling molecules that contain SH2 domains and bind to the LepRb–JAK2 complex. This process subsequently triggers the phosphorylation of the signaling proteins by JAK2, which, in turn, transmits leptin-specific signals to second-order neurons situated in the hypothalamic nucleus [40].

Leptin and its receptor are important pharmacological targets because an excess, deficiency, and resistance to leptin are linked to various human pathologies, including obesity, fertility, cancer, and autoimmune illnesses [39,40,41]. The decrease in leptin concentrations after fasting is responsible for the starvation-induced suppression of the hypothalamic–pituitary–gonadal axis, as well as the malfunction of several other neuroendocrine axes. Therefore, leptin appears crucial in mediating the relationship between adipose tissue, hypothalamic centers regulating energy homeostasis, and the reproductive system [35,38].

Leptin acts as a suppressor on neuropeptide Y (NPY), a potent appetite stimulator; it is known to regulate various pituitary hormones, e.g., suppression of growth hormone (GH) through stimulation of somatostatin, suppression of gonadotropins, or stimulation of the pituitary–adrenal axis [36,37].

Cortisol has been shown to stimulate leptin production and can induce leptin resistance [37]. At the same time, leptin production is positively correlated with insulin concentration [36,37].

Growth hormone has a negative feedback loop with leptin and stimulates the production of growth hormone-releasing hormone [9,39,41]. 

#### 1.2.2. Obesity and Leptin

Different studies show a strong positive connection between serum leptin levels and the percentage of body adiposity [42,43,44]. The clinical characteristics of extreme obesity, decreased satiety, intense hyperphagia, persistent food-seeking behavior, recurrent bacterial infections, hyperinsulinemia, liver steatosis, dyslipidemia, and hypogonadotropic hypogonadism are the outcomes of a complete leptin deficit.

Leptin resistance, or resistance to the anorectic and body-weight-reducing effects of leptin, is linked to hyperleptinemia. Common obesity is characterized by leptin resistance and hyperleptinemia [37]. Forms of leptin have been created and investigated to reverse obesity, but promising effects were seen only in leptin-deficient pathologies. 

Leptin regulates glucose metabolism centrally and peripherally, and its action depends on the degree to which insulin signaling pathways are activated.

Both central and peripheral leptin have the favorable effect of reducing ectopic fat deposition, which is helpful in illnesses linked to obesity [44].

Leptin’s peripheral target tissues include the endocrine pancreas, liver, skeletal muscle, adipose tissues, immune cells, and cardiovascular system. Leptin is present in all types of fat depots. Circulating leptin levels are positively related to obesity in lean humans and animals. Extended fasting is linked to a rapid decrease in plasma leptin levels, which regulates appetite [45]. 

Some studies conducted many years ago on rats showed that lesions in the arcuate nucleus, ventromedial hypothalamus nucleus (satiety center), or dorsomedial hypothalamus nucleus could cause hyperphagia and obesity in rats. In contrast, lesions of the lateral hypothalamic nuclei (hunger center) can cause hypophagia [46]. 

Subsequent research has shown that leptin can stimulate brain pathways targeted by inhibitors to suppress appetite and inhibit neural pathways activated by appetite stimulants in order to reduce calorie intake. Agouti-related protein (AgRP) and neuropeptide NPY are examples of neuropeptides that stimulate appetite. Hunger is inhibited by alpha-melanocyte-stimulating hormone (α-MSH), produced by proopiomelanocortin (POMC). Neurons in the central melanocortin system involved in regulating energy balance are among those that express AgRP, POMC, and melanocortin [47,48,49]. 

Obese individuals present pathologically elevated circulating leptin as a sign of leptin resistance. This refers to the diminished sensitivity of the brain or inability to respond to leptin, indicating a decline in the capacity of leptin to reduce appetite or increase energy expenditure. This, in turn, leads to an increase in food intake and the eventual development of overweight, obesity, cardiovascular disease, and other metabolic disorders [49]. 

## 2. Methodology

**Figure 1:**
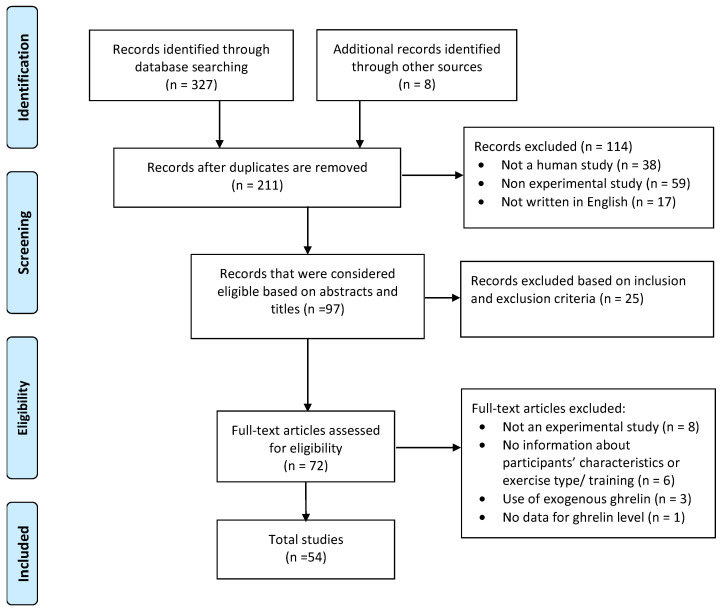
PRISMA flow diagram.

A thorough systematic review was performed and reported using the Preferred Reporting Items for Systematic Reviews and Meta-Analyses (PRISMA) criteria [Figure 1]. Research assessing the effect of physical activity on leptin and ghrelin levels was acceptable for inclusion.

### 2.1. Inclusion Criteria 

Studies were considered for inclusion if they met the following requirements: (1) written in English, and published in a peer-reviewed journal; (2) included individuals of any gender, age, weight status, level of physical fitness, and health; (3) used physical exercise as a standalone intervention or in conjunction with other interventions; (4) applied resistance or endurance training using exercises of varying kinds, intensities, and durations; and (5) included a minimum of two measurements (pre- and post exercise/training) of ghrelin, regardless of the form found. 

The following criteria were used to rule out studies: (1) reviews, case reports, comments, opinions, or editorials; (2) they applied an intervention without any physical exercise; (3) they did not provide information about the type, intensity, frequency, or duration of the exercise or training; (4) they used exogenous ghrelin administration; or (5) they involved animals.

### 2.2. Literature Search Strategy and Study Selection

From 1 January 2009 to 1 February 2024, research was conducted across several significant databases, including PubMed, Scopus, EBSCO Host, Google Scholar, Academic Search Premier, ScienceDirect, and Springer-Link. Using the operators “AND”, “OR”, and “ghrelin” OR “leptin” OR “appetite-related peptides” OR “gastrointestinal peptides” OR “gastrointestinal hormones”, the following essential phrases were added and combined: (“exercise” OR “acute exercise” OR “chronic exercise” OR “training” OR “physical activity” OR “endurance training” OR “resistance training”). In addition, relevant studies were found in the full-text publications’ reference lists and by searching related articles and citations in the PubMed database.

A systematic review (rather than a meta-analysis) was conducted because of the significant heterogeneity of the included studies with respect to the type of ghrelin found, participant characteristics, and activity.

### 2.3. Results

The literature search found 327 records. After screening complete texts, abstracts, and titles, 54 pertinent papers were identified and added to the final analysis (Figure 1 and Supplement Table S1). 

#### 2.3.1. Selected Studies

Based on the inclusion and exclusion criteria, two authors (M.B.I. and R.N.) independently assessed the titles retained from the abovementioned literature search. Potentially relevant citations were filtered down to the abstract level. Full-text papers were evaluated where the abstracts suggested that they should be included. Relevant data, such as sample size, participant characteristics (e.g., sex, age class, body mass phenotype, level of fitness/training, health condition), exercise modality (e.g., acute, chronic), type (e.g., aerobic, resistance, intermittent, combined), intensity (e.g., moderate, intense), duration, and analytical characteristics (e.g., ghrelin form detected, method of analysis, precision), were extracted for each eligible study. The quantity and direction of changes in circulating ghrelin were the primary endpoints. Changes in body weight/fat and circulating growth hormone were also collected when available. Any differences in opinion between the two authors about the choice of studies or the data extraction process were settled by discussion and agreement between all of the authors.

#### 2.3.2. Participants Characteristics

Of the 54 included studies, 33 looked at the ghrelin and leptin responses to acute activity, and 21 looked at the responses to chronic exercise. The trials varied greatly with respect to the kind, frequency, intensity, and length of exercise/training. Multiple exercise types, intensities, and durations were used in numerous trials: aerobic (n = 20), resistance (n = 18), intermittent (n = 8), or combination (n = 8). In terms of time, 27 studies, 21 studies, and 6 studies used exercise for less than 45 min, more than 45 min, and more than 90 min, respectively.

#### 2.3.3. Exercise Type

The ghrelin and leptin responses to acute activity were studied in 33 of the 54 included studies, and the reaction to chronic exercise was studied in 21 studies. The trials varied greatly with respect to the kind, frequency, intensity, and length of exercise/training. Multiple exercise types, intensities, and durations were used in various trials. Aerobic (n = 13), resistance (n = 9), intermittent (n = 5), or combination (n = 6) exercise was utilized in the acute exercise investigations. Regarding time, 15, 11, and 7 studies used exercise for less than 45 min, more than 45 min, and more than 90 min, respectively. Progressive time training was implemented in six research studies. 

Studies on chronic exercise included training programs that were either aerobic (n = 8), resistance (n = 7), a combination (n = 4), or intermittent (n = 2). The majority of these studies (n = 25) employed a long-term program (≥12 weeks), while six studies employed very long-term (>48 weeks) training programs. The majority of research on acute or chronic exercise involved moderately intense exercise. A few studies paired physical training with other therapies, such as dietary interventions [22,50,51,52].

This review also included the long-term effects of specific sports, such as cycling, swimming, and yoga [27,30,53,54,55,56]. 

Table 1 briefly features the included studies, the number and status (obese/overweight) of participants, and training results on ghrelin and leptin levels.

## 3. Exercise-Induced Changes in Ghrelin and Leptin 

### 3.1. Effect of Exercise on Ghrelin

Since exercise increases growth hormone and decreases ghrelin concentrations in the bloodstream (due to blood redistribution, sympathetic nervous system activity, gastrointestinal motility, cytokine release, free fatty acid concentrations, lactate production, and changes in plasma glucose and insulin concentrations), most investigations started with the premise that exercise will suppress ghrelin [78,96,97]. Acute exercise has conflicting effects on ghrelin [Table 1]. However, other research suggests that the sensation of hunger temporarily decreases during and shortly after exercise, a phenomenon known as “exercise-induced anorexia” [17].

In endurance training, ghrelin levels increase due to decreased fat loss and weight loss. However, it has been shown that reducing oxidative stress stimulates ghrelin secretion. Exercises also have an impact on oxidative capacity, reducing oxidative stress levels. The possible mechanisms by which weight loss increases circulating ghrelin are (1) an increase in carbohydrate use, regardless of growth hormone; (2) anabolic effects as a consequence of GH increase and insulin-like growth factor-1; and (3) stimulating food intake via neuropeptide Y [50,98].

Several investigations have shown that short-term exercise, regardless of exercise intensity or type, has little effect on plasma ghrelin levels during or after exercise in participants who are severely obese or overweight [27,29,30,59,60,63,78,87,88,91,95,99,100,101]. However, following exercise, there is a significant drop in the hypothalamic concentration of ghrelin [8]. Furthermore, studies have shown that total and acylated ghrelin levels significantly increase during and after exercise [53,74]. These studies have also shown that this increase is more significant in subjects of average weight than in obese subjects, and that it occurs more during fasting rather than feeding [85,102].

It has been demonstrated that acute aerobic exercise (AEx) reduces the expected increase in hunger and energy intake brought on by a calorie deficit from an increased energy expenditure [29,63]. Changes in gastrointestinal peptides—such as elevated peptide YY and glucagon-like peptide-1, or reduced ghrelin—may regulate this. However, exercise may also promote the regulation of appetite and energy intake by affecting non-homeostatic processes, including food cravings and subjective hunger [29,68,82]. For example, studies using AEx have demonstrated decreased neural responses in brain areas linked to food reward circuits, and these responses are frequently followed by decreased appetite and enhanced satiety [29,63,82]. Conflicting findings from chronic AEx therapies have been seen, with moderate or no impact on appetite-related indicators (such as gut peptides and self-reported appetite) [61,63,83,103].

Halliday T.M. et al. published a study in 2021 focusing on the effects of acute REx (resistance exercise), in contrast with acute AEx (aerobic exercise), on reductions in both orexigenic (ghrelin) and anorectic (PYY and GLP-1) gut peptides. In both exercise conditions, the ad libitum energy intake did not rise in comparison with SED (the sedentary control group), suggesting that both exercise modalities have effects that inhibit hunger and energy intake [29]. 

A few years earlier, Tobin S.Y. et al. published a study on 24 individuals with obesity/overweight who fulfilled three requirements: (1) AEx (45 min at 65–70% of the age-predicted maximal heart rate), (2) REx (one set to failure on 12 exercises), and (3) CON. Every scenario started 35 min after a standardized breakfast or postprandial. Hormones (ghrelin, PYY, and GLP-1) and appetite (visual analog scale for hunger, satiety, and prospective food consumption [PFC]) were evaluated while fasting and every 30 min postprandially for three hours. At lunch, post-exercise ad libitum energy intake was also assessed. The results indicated that although women have comparable appetites to males after an acute exercise session or period of inactivity, they reported lower ratings of hunger after such events [63]. 

Gender analysis in response to appetite and exercise was the subject for Hagobian et al. [95]. They analyzed the sex differences in how energy regulates hormones and appetite perception in response to exercise. Eighteen overweight and obese people performed four exercise sessions with energy added to their baseline diet, in order to maintain an energy balance (BAL), and four without energy supplied to their baseline diet, in order to induce an energy deficit (DEF). The findings suggest that exercise, independent of calorie status, changed hormones that regulate energy intake in women in a way that was predicted to increase energy intake. When the energy balance was maintained, the reaction to exercise in males was eliminated [95]. The statistics support the hypothesis that women’s strategies for controlling body fat are more successful [78,87].

In addition to gender, it seems that an obesity-associated gene (FTO), the rs9939609 A-allele, is connected to obesity, increased caloric consumption, and higher concentrations of acyl-ghrelin (AG); nevertheless, the risk of obesity associated with this allele may be reduced by exercise. Des-acyl-ghrelin (DAG) is produced when butyrylcholinesterase (BChE) hydrolyzes AG, which may reduce appetite. Exercise and the FTO rs9939609 genotype may have unknown effects on BChE activity, AG, DAG, and calorie intake. Exercise corrects the increased AG profile in AA people at risk of obesity, raises BChE activity, and lowers AG and the AG/DAG ratio. These results imply that BChE activity-targeting exercises or other approaches could provide AA people with a therapeutic or preventive approach [69].

The most common type of exercise training used in research is walking. The conclusions on those who are overweight or obese are ambiguous. Unick et al. implied that walking did not affect hunger or energy intake, indicating that people who are overweight or obese do not acutely compensate for the energy expenditure of the exercise session by consuming more calories [99]. This allows an energy deficit to continue after exercise, which may support weight control.

The impact of walking ambient conditions on energy intake (EI) was also investigated. After exercising in the cold, EI was considerably higher (1299 ± 657 kcal (mean ± SD)) than after exercising in a neutral setting (1172 ± 537 kcal (mean ± SD)) (*p* < 0.05). There was a significant difference in the acylated ghrelin concentrations and AUC values (*p* < 0.05) when walking in the cold as opposed to when walking in the neutral setting [91]. These results indicate that exercise in the cold increases post-exercise emotional intelligence (EI) in overweight people more than in a neutral setting.

Swimming is an excellent beneficial exercise for those with obesity and arthritis, since it involves less heat and little weight bearing. Nevertheless, some old convictions suggest that daily swimming might not help lose weight and body fat. It is currently thought that working out in cold water increases hunger. Figo et al. examined these elements in their study “Swimming and Cycling Exercise Interventions in Adults with Obesity”. The swimming or cycling exercise training did not affect the fasting plasma concentrations of ghrelin, insulin, leptin, or peptide YY (*p* > 0.05). In persons with obesity and osteoarthritis, swimming exercise did not adversely affect hormones associated with hunger that might hinder weight loss [54].

In research where subjects engaged in long-term physical activity, ghrelin levels increased due to reduced body fat and weight reduction [9,34,55,66]. Research has demonstrated increasing ghrelin secretion with decreasing oxidative stress [9,34,100,101]. Additionally, it is well recognized that exercise training significantly improves oxidative capacity and reduces reactive oxygen stress levels [34,53,83]. Although the exact process for weight reduction to increase circulating ghrelin levels is unknown, three systems are likely to be involved in the stimulation of positive energy balance: (1) Regardless of GH, a decrease in fat consumption and increased carbohydrate utilization. (2) The anabolic impact is brought on by increased GH and IGF-1 synthesis. (3) The use of neuropeptide Y (NPY) to stimulate long-term food intake.

For 12 weeks of aerobic activity, with three sessions per week, four groups of eleven people were randomly selected from among forty-four overweight men: (1) an endurance group (three sets of 10 min at 80–90% of maximum heart rate), (2) a resistance group (four sets of eight repetitions at 80% of one-repetition maximum), (3) a concurrent group (combination of endurance and resistance group programs in alternating order), and (4) a control group. The findings demonstrated that all three types of training regimens reduced BMI (*p* = 0.000, *p* = 0.000, and *p* = 0.034), decreased serum acylated ghrelin (*p* = 0.000, *p* = 0.000, and *p* = 0.004), and elevated PYY hormone levels (*p* = 0.000, *p* = 0.000, and *p* = 0.036 for the resistance, concurrent, and endurance groups, respectively). Resistance exercise, however, had a more noticeable impact on these modifications. Furthermore, the blood levels of GLP-1 were unaffected by all of the exercise regimens. Moreover, weight (*p* = 0.003) and BMI (*p* = 0.009) changes with ghrelin showed a significant positive association, but the weight (*p* = 0.003) and BMI (*p* = 0.03) changes with PYY showed a negative correlation. According to the literature, regular exercise—especially resistance training—may help sedentary overweight individuals lose weight and improve their body composition by increasing anorexigenic hormones and reducing orexigenic hormones [65].

Among obese teenagers, there was a significant decrease in body weight during the lifestyle modification, without any considerable change in peptide YY (PYY). This was correlated with an apparent increase in ghrelin (from month 6; *p* < 0.05), a decrease in leptin (from month 3; *p* < 0.05), and decreases in insulin and HOMA (from month 3; *p* < 0.0001). No correlation was seen between anthropometric alterations and changes in ghrelin and PYY following the lifestyle adjustment, nor with baseline ghrelin levels. Higher anthropometrical alterations were, however, connected with a higher baseline PYY (*p* < 0.1). Long-term weight loss and elevated ghrelin concentrations were seen in teenagers with severe obesity who received supervision during their aerobic exercise regimen and followed a balanced diet, without affecting their PYY concentrations. Furthermore, it is possible to think of baseline PYY concentrations as indicators of weight reduction [31].

Markofski et al. found that a 12-week aerobic and resistance training program increased fasting ghrelin concentrations by 47% in 70-year-old individuals [104]. Interestingly, recent results suggest that the effect of exercise training on ghrelin concentration might depend on the volume of exercise. Four months of a moderate dose of aerobic exercise favored reduced ghrelin, while ghrelin levels remained unchanged in response to a low-dose training program in older women [8,51,105].

Yoga training for a year improved physical functioning and central adiposity in persons with MetS while modifying total ghrelin, UnAG, AG, obestatin, and GH. Yoga’s health benefits might be linked to GH and the ghrelin gene product changes [53]. 

### 3.2. Effect of Exercise on Leptin 

Leptin is one hormone that regulates energy balance and metabolism, influencing body weight. Leptin reduces appetite and increases energy expenditure through its effects on the hypothalamus and other parts of the central nervous system [2,57,65]. Moreover, it is well known that leptin is positively correlated with body mass index (BMI) and other indicators of obesity [47,62,67].

Thus far, research has demonstrated that physical activity and training have varying impacts on leptin levels [Table 1], exhibiting both stimulatory and inhibitory effects [54,58,64,70,94].

In a previous study, male patients with stable coronary heart disease were randomized to aerobic training three times a week at 60–80% of their maximal heart rate, or to a control group. There were no significant changes in leptin levels after training. Still, the values were increased in the control group [94]. 

Another research team that studied 80 young males noted the same tendency of leptin to preserve its levels after training. The subjects had a standard nutrient beverage and, after 1.5 h, initiated a 90 min treadmill exercise. The leptin levels increased initially, but after one hour of recovery, they decreased again [92].

Later, Sartor F. et al. compared two groups of obese adults after a CHO-reduced diet combined with an energy-restricted diet in one group and a diet combined with high-intensity interval training (HIIT) carried out 10 times (three times per week) in the other [93]. Oral glucose insulin sensitivity increased significantly in both groups, the fasting respiratory exchange ratio decreased significantly in both groups, and the leptin level decreased significantly in both groups.

Some studies conducted on young adults assessed the impact of moderate-intensity and prolonged exercise on appetite hormones and showed lower plasmatic levels of leptin and increased cortisol [84,90]. Researchers using moderate–high-intensity prolonged exercises did not detect any important changes in acylated ghrelin and/or leptin levels (depending on the exercise volume) [78]. They found no significant changes in the training order influencing the appetite-regulating hormones between subjects following different types of training [90].

Many research teams have decided to include sex-related subjects in the discussion, as it is known that sex influences the effects of exercise on different hormones. Guadalupe-Grau A. et al. followed the results of nine weeks of combined strength and plyometric training on osteocalcin and leptin in two different groups composed of 43 males and 23 females both pre- and post training [106]. The findings suggest a negative correlation of osteocalcin with leptin, fat mass, and body fat percentage. After training, the positive impact of physical activity on performance, muscle hypertrophy, and osteogenesis was similar between the groups, while for the females, the serum leptin was significantly lower. 

Taken independently, both males and females in different studies had lower leptin plasma levels after training [71,77]. 

Overweight or obese subjects may have a slower reaction pattern regarding serum concentrations of leptin, as it is well known that leptin resistance is increased in these cases [40,42,47]. Bjersing et al. studied 43 females following 15 weeks of twice-weekly training and concluded that leptin levels were reduced in lean women but not in overweight and obese ones [75]. Still, different groups of researchers have all studied overweight or obese people and concluded that leptin concentrations decrease after exercise [62,66,67,72,73,81,84,90,93,106]. 

Morishma et al. conducted a four-week study in both hypoxic and normoxic training conditions and found similar effects, with increased plasma ghrelin and decreased leptin. [80]. Mendham et al. compared leptin changes after small-sided games versus cycling training in a three-days-per-week eight-week study and found that only small-sided game training effectively reduced leptin [86]. Salvadori et al. showed a higher reduction in leptin concentration in the aerobic training group after four weeks of aerobic versus aerobic with a bout of anaerobic training [79]. Racil et al. compared the effects of 12-week high-intensity interval training (HIIT) with the effect of a 12-week plyometric exercise combined with HIIT. They found that both groups had reduced leptin levels [76]. Still, the combined program had a greater impact on leptin levels, suggesting that it was more efficient. Caldeira et al. carried out a five-week study, three times per week, to compare the effects of HIIT and steady-state training (SST) on leptin and soluble leptin receptor (sOB-R) levels [70]. Leptin was lower at the end of both training groups, but sOB-R was increased only after HIIT, showing a greater impact on the central response.

Ahmadizad et al. followed a study using eight-week resistance training (RT) composed of either non-periodized or periodized training [89]. They concluded that there were no differences between groups regarding the effect of reducing leptin levels. However, short-term periodized RT improves overweight men’s insulin resistance and muscular strength. Middelbeek et al. designed a study using short-term exercise programs with either moderate-intensity training (MIT) or sprint-intensity training (SIT) [64]. They found similar results in reducing leptin after and improving cardiovascular fitness.

## 4. Discussion

As previously mentioned, ghrelin secretions may be influenced by exercise intensity through catecholamine-related mechanisms [7]. However, no research has explored how ghrelin reacts to intense exercise or training, which is known to increase catecholamine secretions in both healthy and obese people. In fact, moderate-intensity training increased acylated ghrelin levels in trained women, according to Larson-Meyer et al. [101]; however, other research has reported that the same training decreased or even did not affect acylated ghrelin levels [33,54,58,63,78,85]. Perhaps the primary reason for these differences is the various training regimens used in these investigations (e.g., training length, type of activity, and intensity).

The degree to which body weight varies is another mechanism that could explain reactions to exercise. It has been noted that, in obese people, exercise-induced weight loss is linked to gradual elevations in ghrelin plasma levels. Nevertheless, these modifications occur when the body weight drops by over 3 kg [51,80]. According to these results, ghrelin may increase after exercise training as a compensatory mechanism for weight loss, but not hyperphagia [53,90]. Furthermore, it has been observed that increases of this kind brought on by diet or exercise training are linked to a decline in central obesity and, in the case of overweight or obese participants, a return to baseline following weight stabilization [25,44,53].

In support of this, a study found that even in participants of normal weight, exercise training or diet-induced weight loss is linked to an increase in ghrelin, which is inversely correlated with actual weight loss [107]. Therefore, we might suggest that modifications to this hormone that regulates body weight act as a compensatory strategy to keep body weight stable. It diminishes fat in individuals, stifles hunger, and ultimately leads to weight loss. On the other hand, in normal-weight subjects, it promotes appetite and weight gain [96]. More research is required to address this issue and offer proof to either confirm or deny this inference.

Another study demonstrated that diet restriction increased acylated ghrelin and lowered PYY3–36 as a compensatory strategy, but exercise with the same energy losses had no effect on these hormones [108]. Thus, these results support the theory that some metabolites, such as glucose, or other hormones, such as insulin and leptin, may influence ghrelin [108].

Exercise training has been shown to reduce leptin levels in obese people, which makes sense given leptin’s function in obesity [42,49]. Increases in leptin sensitivity or its turnover (i.e., hormonal elimination) that reduce fat mass may be the processes underlying this reduction [37]. Furthermore, following exercise training, increases in leptin levels are more strongly connected with changes in body fat percentage than with changes in body mass index [39,71]. Additionally, research suggests that exercise intensity is significant in regulating how leptin responds to exercise training [70,71,84].

While the exact mechanism behind leptin’s ability to suppress appetite is unknown, research has demonstrated that leptin’s induction of AMPK in the hypothalamus results in acetyl-COA carboxylase (ACC) inactivation [35,37].

According to news reports, body weight increases upon inactivating malonyl-COA carboxylase, which validates this finding [109,110]. ACC’s malonyl-COA substrate suppresses carnitine palmitoyltransferase (CPT) to prevent fat oxidation [110]. These results demonstrate malonyl-COA’s important function in controlling hunger. Furthermore, leptin selectively increases malonyl-COA concentrations by activating ACC, which may obstruct CPT1-a in the hypothalamus [37,109]. Nevertheless, there is a theory that CPT1-c might modulate the hypothalamic leptin anorectic signaling pathway [110,111].

Excessive activity has been linked to hunger suppression during the recovery phase. Additionally, research shows that while moderate continuous activity and sprint interval training can increase GLP-1 and reduce feelings of hunger, they do not affect PYY. Additionally, the suppression of hunger sensations lasts longer following sprint interval exercises, which may be connected to the elevated plasma glucose levels seen following intense exercise [30,61]. It has been demonstrated that elevated plasma glucose levels are linked to malonyl-COA in the hypothalamus, lending credence to this theory. Increased brain glucose inflow may cause this action, since it raises malonyl-COA, decreases the AMP/ATP ratio, inactivates AMPK, and ultimately raises ACC activity [37,111].

Despite many recent studies, there is still a debate over the exact mechanism by which exercise training reduces leptin and suppresses appetite.

Exercise training has enhanced skeletal muscle’s lipid (fat) oxidation capability by reducing malonyl-COA levels and ACC activity [62,76,110]. Given that malonyl-COA plays a major part in the brain’s signals that regulate appetite, exercise training may have the opposite impact in the hypothalamus. We can only hypothesize, however, because there is no research on this in humans. It is possible that exercise training increases malonyl-COA and prematurely suppresses food intake by boosting fat oxidation in peripheral tissues and maintaining plasma glucose levels. Future studies should clarify these assumptions.

Still, to date, there is no firm evidence supporting the use of leptin as a pharmacological therapy [112,113,114,115,116,117,118,119,120,121,122]. We know that physical exercise reduces leptin, but the effect is stronger in lean subjects than in overweight or obese ones [71,84,114,115,123,124,125]. Further research should concentrate on finding novel pathways of whole-body leptin regulation to develop new medications that counteract leptin resistance. In this sense, knowledge of the pathophysiology of obesity-related illnesses and the role that leptin plays in regulating energy balance should lead to the development of novel therapeutic options for obesity.

**Figure 2:**
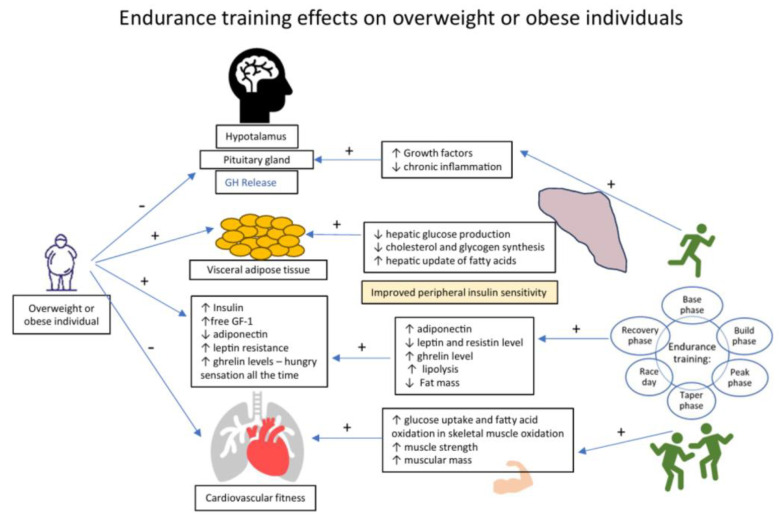
Endurance training’s effects on overweight or obese individuals.

Figure 2 summarizes the impact of physical exercise on the main areas and mechanisms involved in appetite regulation among overweight or obese subjects.

### 4.1. Strengths

All of the selected studies have specific methodologies regarding ghrelin and leptin measurements and include quantitative data that are consistent, precise, and reliable. The training is well designed and described, and different types of training are sometimes compared in order to obtain the finest results.

### 4.2. Limitations

Some of the selected studies had small sample sizes, so the data are not robust enough to explain the complex topic of hunger and satiety, and not all of the studies obtained significant results.

In addition to ghrelin and leptin as major hormones regulating appetite, there are other factors that balance food intake and metabolism, such as GLP1, PYY, and adiponectin, but their measurement was not included as elements in this review. Insulin, glucagon, and different medications with different metabolic impacts were not specific items to be highlighted, although they also influence appetite.

Further research should concentrate on determining the complex relationships between all of the factors influencing appetite in connection to physical exercise. This would be interesting to see, especially if antidiabetic or antimetabolic disease drugs impact the intensity level of exercise training.

## 5. Conclusions

In summary, the majority of these hormones associated with appetite are affected by the levels of activity in both healthy and overweight people. Findings on obese people are still scarce and occasionally inconsistent; nevertheless, many of these hormones have undergone in-depth research. For instance, leptin’s functions and the impacts of stimulating substances have been thoroughly studied, whereas other aspects have only been partially and unevenly studied.

Therefore, more investigation is needed to clarify how these hormones affect appetite and decrease hunger in obese or severely obese people, in order to validate the efficacy of exercise training. Furthermore, additional research is required to examine the benefits of novel exercise training regimens, including concurrent exercise training, functional training, cognitive therapy, high-intensity interval training (HIIT), aquatic training, and functional training, or various combinations of these training techniques. Do these methods have an impact on these hormones, and are they successful in altering them? There is currently no conclusive response to this topic.

Finally, given that men and women differ in the hormones linked to obesity, examining the impact of gender on acute reactions and adaptations to exercise training would be extremely beneficial and offer insightful data for prescribing exercise to various groups.

## Figures and Tables

**Table 1:** Exercise-induced changes in ghrelin and leptin.

Study (Reference)	Participants (Number)	Results
Najafi R et al. [52]	30 obese girls	Leptin↑; ghrelin↓
Oh DH, Lee JK et al. [57]	16 obese adults	Leptin↓
Heinen D et al. [58]	105 adults with overweight/obesity	Leptin↑
Li S, Guo R et al. [59]	14 obese adults	↓appetite; ghrelin↓
Rostamzadeh, N et al. [60]	30 obese adults	Acylated ghrelin↓
Beer NJ et al. [61]	36 adults	↓appetite
Murawska-Cialowicz E. et al. [62]	75 with overweight/obesity	Leptin↓
Halliday TM et al. [29]	24 overweight adults	ghrelin↓
Tobin SY et al. [63]	24 adults with overweight/obesity	ghrelin↓; ↓appetite
Middelbeek RJW et al. [64]	22 sedentary males	Leptin↓
Fico BG et al. [54]	39 adults with obesity and osteoarthritis	No significant effect
Shakiba E et al. [65]	44 overweight adults	↓acylated ghrelin
Ouerghi et al. 2019 [8]	7 overweight adults	No significant effect
Heiston et al. [28]	28 obese adults	No significant effect
Quist JS et al. [66]	130 adults with overweight/obesity	acylated ghrelin↑
Tremblay A, et al. [67]	100 overweight adults	ghrelin↑ at 3 months; no change at 6 months
Liao J et al. [68]	16 obese children	ghrelin↑
Dorling JL et al. [69]	24 adults with obesity-linked polymorphism	Acylated ghrelin↓
Yu AP et al. [53]	79 obese adults	Acylated ghrelin↓
Caldeira RS et al. [70]	20 overweight/obese adults	Leptin↓
Inoue D et al. [71]	16 overweight/obese adults	Leptin↓
Kang SJ et al. [55]	13 obese females	Leptin↓ ghrelin↑
Jackson M et al. [72]	70 overweight/obese adults	Leptin↑; ghrelin↓
Vardar S, et al. [73]	12 overweight/obese females	Leptin↓
Holliday A. et al. [30]	8 overweight adults	Acylated ghrelin↓
Martins C et al. [74]	45 obese adults	No significant effect
Bjersing JL et al. [75]	43 overweight females	Leptin↓
Racil G et al. [76]	68 obese females	Leptin↓
Tan S. et al. [77]	30 overweight individuals	Leptin↓
Douglas JA et al. [78]	15 overweight males	No significant effect
Gibbons et al. [56]	32 overweight/obese individuals	Leptin↑; ghrelin↓
Martins C et al. [27]	12 overweight/obese adults	Acylated ghrelin↓
Mason C et al. [51]	439 overweight or obese females	ghrelin↑
Salvadori A. et al. [79]	12 overweight/obese females	Leptin↓
Morishima, T. et al. [80]	20 overweight/obese adults	Leptin↓
Damaso AR. Et al. [81]	139 obese individuals	Leptin↓
Gholipour M et al. [82]	8 overweight adults	Acylated ghrelin↓; ↓appetite
Sim AY et al. [83]	17 overweight men	ghrelin↓
Campos RM et al. [32]	42 obese adolescents	ghrelin↑; leptin↓
Zaccaria M, et al. 2013 [84]	7 overweight males	leptin↓
Heden TD et al. 2013 [85]	14 obese females	No significant effect
Mendham A. et al. [86]	33 overweight/obese males	leptin↓
Tiryaki-Sonmez G et al. [87]	9 overweight females	No significant effect
Karacabey K. et al. [88].	40 obese boys	leptin↓
Ahmadizad S et al. [89]	32 overweight/obese males	No significant effect
Jones TE et al. [33]	12 overweight adolescents	No significant effect
Gueugnon C et al. [31]	32 obese inactive adolescents	ghrelin↑; leptin↓
Loria-Kohen V et al. 2012 [90]	119 overweight individuals	leptin↓
Thomas GA et al. [91]	19 obese men	ghrelin↑
Kraemer R et al. 2011 [92]	80 overweight/obese males	leptin↓
Martin C et al. [74]	22 overweight/obese adults	ghrelin↑
Sartor F et al. [93]	19 obese adults	leptin↓
Kosydar-Piechna M. et al. [94]	64 overweight males	No significant effect
Hagobian TA et al. [95]	9 overweight males	ghrelin↑

↑—increased; ↓—decreased.

## Supplementary Materials

The following supporting information can be downloaded at https://www.mdpi.com/article/10.3390/ijms25158067/s1.

## References

[1] Yeung A.Y., Tadi P. (2024). Physiology, Obesity Neurohormonal Appetite and Satiety Control. StatPearls.

[2] Hill J.W., Faulkner L.D. (2017). The Role of the Melanocortin System in Metabolic Disease: New Developments and Advances. Neuroendocrinology.

[3] Giordano A., Nisoli E., Sbraccia P., Finer N. (2019). Neuroendocrinology of Energy Balance. Obesity: Pathogenesis, Diagnosis, and Treatment.

[4] Sasaki T. (2017). Neural and Molecular Mechanisms Involved in Controlling the Quality of Feeding Behavior: Diet Selection and Feeding Patterns. Nutrients.

[5] Vigil P., Meléndez J., Petkovic G., Del Río J.P. (2022). The Importance of Estradiol for Body Weight Regulation in Women. Front. Endocrinol..

[6] Kojima M., Hosoda H., Date Y., Nakazato M., Matsuo H., Kangawa K. (1999). Ghrelin Is a Growth-Hormone-Releasing Acylated Peptide from Stomach. Nature.

[7] Kojima M., Kangawa K. (2005). Ghrelin: Structure and function. Physiol. Rev..

[8] Ouerghi N., Feki M., Bragazzi N.L., Knechtle B., Hill L., Nikolaidis P.T., Bouassida A. (2021). Ghrelin Response to Acute and Chronic Exercise: Insights and Implications from a Systematic Review of the Literature. Sports Med..

[9] Algul S., Ilcin S., Ozcelik O. (2021). Effects of Excercise on Ghrelin. Prog. Nutr..

[10] Labarthe A., Tolle V. (2016). Ghrelin: A gastric hormone at the crossroad between growth and appetite regulation. Biol. Aujourd’hui.

[11] Hassouna R., Labarthe A., Tolle V. (2016). Hypothalamic Regulation of Body Growth and Appetite by Ghrelin-Derived Peptides during Balanced Nutrition or Undernutrition. Mol. Cell. Endocrinol..

[12] Smitka K., Prochazkova P., Roubalova R., Dvorak J., Papezova H., Hill M., Pokorny J., Kittnar O., Bilej M., Tlaskalova-Hogenova H. (2021). Current Aspects of the Role of Autoantibodies Directed Against Appetite-Regulating Hormones and the Gut Microbiome in Eating Disorders. Front. Endocrinol..

[13] Perelló M., Dickson S.L., Zigman J.M., Leggio L. (2023). Toward a Consensus Nomenclature for Ghrelin, Its Non-acylated Form, Liver Expressed Antimicrobial Peptide 2 and Growth Hormone Secretagogue Receptor. J. Neuroendocrinol..

[14] Devesa J. (2021). The Complex World of Regulation of Pituitary Growth Hormone Secretion: The Role of Ghrelin, Klotho, and Nesfatins in It. Front. Endocrinol..

[15] Lewiński A., Karbownik-Lewińska M., Wieczorek-Szukała K., Stasiak M., Stawerska R. (2021). Contribution of Ghrelin to the Pathogenesis of Growth Hormone Deficiency. Int. J. Mol. Sci..

[16] Deschaine S.L., Leggio L. (2022). From “Hunger Hormone” to "It’s Complicated: Ghrelin Beyond Feeding Control. Physiology.

[17] Howick K., Griffin B.T., Cryan J.F., Schellekens H. (2017). From Belly to Brain: Targeting the Ghrelin Receptor in Appetite and Food Intake Regulation. Int. J. Mol. Sci..

[18] Nartea R., Mitoiu B.I., Nica A.S. (2019). Correlation between Pregnancy Related Weight Gain, Postpartum Weight Loss and Obesity: A Prospective Study. J. Med. Life.

[19] Sun J., Tan Y., Su J., Mikhail H., Pavel V., Deng Z., Li Y. (2023). Role and Molecular Mechanism of Ghrelin in Degenerative Musculoskeletal Disorders. J. Cell. Mol. Med..

[20] Cheung C.K., Wu J.C.-Y. (2013). Role of Ghrelin in the Pathophysiology of Gastrointestinal Disease. Gut Liver.

[21] Alamri B.N., Shin K., Chappe V., Anini Y. (2016). The Role of Ghrelin in the Regulation of Glucose Homeostasis. Horm. Mol. Biol. Clin. Investig..

[22] Uchida A., Zechner J.F., Mani B.K., Park W., Aguirre V., Zigman J.M. (2014). Altered Ghrelin Secretion in Mice in Response to Diet-Induced Obesity and Roux-En-Y Gastric Bypass. Mol. Metab..

[23] Atalayer D., Gibson C., Konopacka A., Geliebter A. (2013). Ghrelin and Eating Disorders. Prog. Neuropsychopharmacol. Biol. Psychiatry.

[24] Mills J.G., Larkin T.A., Deng C., Thomas S.J. (2019). Weight Gain in Major Depressive Disorder: Linking Appetite and Disordered Eating to Leptin and Ghrelin. Psychiatry Res..

[25] Van den Houte K., Scarpellini E., Verbeure W., Mori H., Schol J., Masuy I., Carbone F., Tack J. (2020). The Role of GI Peptides in Functional Dyspepsia and Gastroparesis: A Systematic Review. Front. Psychiatry.

[26] Papandreou D., Karavolias C., Arvaniti F., Kafeza E., Sidawi F. (2017). Fasting Ghrelin Levels Are Decreased in Obese Subjects and Are Significantly Related with Insulin Resistance and Body Mass Index. Open Access Maced. J. Med. Sci..

[27] Martins C., Stensvold D., Finlayson G., Holst J., Wisloff U., Kulseng B., Morgan L., King N.A. (2015). Effect of Moderate- and High-Intensity Acute Exercise on Appetite in Obese Individuals. Med. Sci. Sports Exerc..

[28] Heiston E.M., Eichner N.Z.M., Gilbertson N.M., Gaitán J.M., Kranz S., Weltman A., Malin S.K. (2019). Two Weeks of Exercise Training Intensity on Appetite Regulation in Obese Adults with Prediabetes. J. Appl. Physiol..

[29] Halliday T.M., White M.H., Hild A.K., Conroy M.B., Melanson E.L., Cornier M.-A. (2021). Appetite and Energy Intake Regulation in Response to Acute Exercise. Med. Sci. Sports Exerc..

[30] Holliday A., Blannin A.K. (2017). Very Low Volume Sprint Interval Exercise Suppresses Subjective Appetite, Lowers Acylated Ghrelin, and Elevates GLP-1 in Overweight Individuals: A Pilot Study. Nutrients.

[31] Gueugnon C., Mougin F., Nguyen N.U., Bouhaddi M., Nicolet-Guénat M., Dumoulin G. (2012). Ghrelin and PYY Levels in Adolescents with Severe Obesity: Effects of Weight Loss Induced by Long-Term Exercise Training and Modified Food Habits. Eur. J. Appl. Physiol..

[32] Campos R.M.S., de Mello M.T., Tock L., Silva P.L., Masquio D.C.L., de Piano A., Sanches P.L., Carnier J., Corgosinho F.C., Foschini D. (2014). Aerobic plus Resistance Training Improves Bone Metabolism and Inflammation in Adolescents Who Are Obese. J. Strength Cond. Res..

[33] Jones T.E., Basilio J.L., Brophy P.M., McCammon M.R., Hickner R.C. (2009). Long-Term Exercise Training in Overweight Adolescents Improves Plasma Peptide YY and Resistin. Obesity.

[34] Wu W., Zhu L., Dou Z., Hou Q., Wang S., Yuan Z., Li B. (2024). Ghrelin in Focus: Dissecting Its Critical Roles in Gastrointestinal Pathologies and Therapies. Curr. Issues Mol. Biol..

[35] Fève B., Bastard J.-P. (2012). From the Conceptual Basis to the Discovery of Leptin. Biochimie.

[36] Yupanqui-Lozno H., Bastarrachea R.A., Yupanqui-Velazco M.E., Alvarez-Jaramillo M., Medina-Méndez E., Giraldo-Peña A.P., Arias-Serrano A., Torres-Forero C., Garcia-Ordoñez A.M., Mastronardi C.A. (2019). Congenital Leptin Deficiency and Leptin Gene Missense Mutation Found in Two Colombian Sisters with Severe Obesity. Genes.

[37] Dornbush S., Aeddula N.R. (2024). Physiology, Leptin. StatPearls.

[38] Montes-de-Oca-García A., Perez-Bey A., Corral-Pérez J., Marín-Galindo A., Calderon-Dominguez M., Velázquez-Díaz D., Casals C., Ponce-Gonzalez J.G. (2023). Influence of Gender on Plasma Leptin Levels, Fat Oxidation, and Insulin Sensitivity in Young Adults: The Mediating Role of Fitness and Fatness. Nutrients.

[39] Cheng J., Luo Y., Li Y., Zhang F., Zhang X., Zhou X., Ji L. (2022). Sex- and body mass index-specific reference intervals for serum leptin: A population based study in China. Nutr. Metab..

[40] Gruzdeva O., Borodkina D., Uchasova E., Dyleva Y., Barbarash O. (2019). Leptin Resistance: Underlying Mechanisms and Diagnosis. Diabetes Metab. Syndr. Obes..

[41] Greco M., De Santo M., Comandè A., Belsito E.L., Andò S., Liguori A., Leggio A. (2021). Leptin-Activity Modulators and Their Potential Pharmaceutical Applications. Biomolecules.

[42] Izquierdo A.G., Crujeiras A.B., Casanueva F.F., Carreira M.C. (2019). Leptin, Obesity, and Leptin Resistance: Where Are We 25 Years Later?. Nutrients.

[43] Landecho M.F., Tuero C., Valentí V., Bilbao I., de la Higuera M., Frühbeck G. (2019). Relevance of Leptin and Other Adipokines in Obesity-Associated Cardiovascular Risk. Nutrients.

[44] Tong Q., Xu Y. (2012). Central Leptin Regulation of Obesity and Fertility. Curr. Obes. Rep..

[45] Chait A., den Hartigh L.J. (2020). Adipose Tissue Distribution, Inflammation and Its Metabolic Consequences, Including Diabetes and Cardiovascular Disease. Front. Cardiovasc. Med..

[46] Huang X.F., Koutcherov I., Lin S., Wang H.Q., Storlien L. (1996). Localization of Leptin Receptor mRNA Expression in Mouse Brain. Neuroreport.

[47] Obradovic M., Sudar-Milovanovic E., Soskic S., Essack M., Arya S., Stewart A.J., Gojobori T., Isenovic E.R. (2021). Leptin and Obesity: Role and Clinical Implication. Front. Endocrinol..

[48] Li M.-D. (2011). Leptin and Beyond: An Odyssey to the Central Control of Body Weight. Yale J. Biol. Med..

[49] Liu J., Yang X., Yu S., Zheng R. (2018). The Leptin Resistance. Adv. Exp. Med. Biol..

[50] Asghari S., Rezaei M., Rafraf M., Taghizadeh M., Asghari-Jafarabadi M., Ebadi M. (2022). Effects of Calorie Restricted Diet on Oxidative/Antioxidative Status Biomarkers and Serum Fibroblast Growth Factor 21 Levels in Nonalcoholic Fatty Liver Disease Patients: A Randomized, Controlled Clinical Trial. Nutrients.

[51] Mason C., Xiao L., Imayama I., Duggan C.R., Campbell K.L., Kong A., Wang C.-Y., Alfano C.M., Blackburn G.L., Foster-Schubert K.E. (2015). The Effects of Separate and Combined Dietary Weight Loss and Exercise on Fasting Ghrelin Concentrations in Overweight and Obese Women: A Randomized Controlled Trial. Clin. Endocrinol..

[52] Najafi R., Heidarianpour A., Shokri E., Shokri B. (2023). Ameliorative Effects of Aerobic Training in Girls with Precocious Puberty: Role of Leptin and Ghrelin. Sci. Rep..

[53] Yu A.P., Ugwu F.N., Tam B.T., Lee P.H., Lai C.W., Wong C.S.C., Lam W.W., Sheridan S., Siu P.M. (2018). One Year of Yoga Training Alters Ghrelin Axis in Centrally Obese Adults with Metabolic Syndrome. Front. Physiol..

[54] Fico B.G., Alkatan M., Tanaka H. (2020). No Changes in Appetite-Related Hormones Following Swimming and Cycling Exercise Interventions in Adults with Obesity. Int. J. Exerc. Sci..

[55] Kang S.-J., Kim J.-H., Gang Z., Yook Y.-S., Yoon J.-R., Ha G.-C., Ko K.-J. (2018). Effects of 12-Week Circuit Exercise Program on Obesity Index, Appetite Regulating Hormones, and Insulin Resistance in Middle-Aged Obese Females. J. Phys. Ther. Sci..

[56] Gibbons C., Blundell J.E., Caudwell P., Webb D.-L., Hellström P.M., Näslund E., Finlayson G. (2017). The Role of Episodic Postprandial Peptides in Exercise-Induced Compensatory Eating. J. Clin. Endocrinol. Metab..

[57] Oh D.-H., Lee J.-K. (2023). Effect of Different Intensities of Aerobic Exercise Combined with Resistance Exercise on Body Fat, Lipid Profiles, and Adipokines in Middle-Aged Women with Obesity. Int. J. Environ. Res. Public Health.

[58] Heinen D., Heissel A., Heinzel S., Fydrich T., Ströhle A., Rapp M.A., Vogel H. (2023). Effect of Acute and Long-Term Exercise on Leptin Levels in Depressed Outpatients. BMC Public Health.

[59] Li S., Guo R., Wang J., Zheng X., Zhao S., Zhang Z., Yu W., Li S., Zheng P. (2023). The Effect of Blood Flow Restriction Exercise on *N*-Lactoylphenylalanine and Appetite Regulation in Obese Adults: A Cross-Design Study. Front. Endocrinol..

[60] Rostamzadeh N., Sheikholeslami-Vatani D. (2022). Appetite Regulating Hormones and Body Composition Responses to Resistance Training and Detraining in Men with Obesity: A Randomized Clinical Trial. Sport Sci. Health.

[61] Beer N.J., Jackson B., Dimmock J.A., Guelfi K.J. (2022). Attenuation of Post-Exercise Energy Intake Following 12 Weeks of Sprint Interval Training in Men and Women with Overweight. Nutrients.

[62] Murawska-Ciałowicz E., Kaczmarek A., Kałwa M., Oniszczuk A. (2022). Influence of Training and Single Exercise on Leptin Level and Metabolism in Obese Overweight and Normal-Weight Women of Different Age. Int. J. Environ. Res. Public Health.

[63] Tobin S.Y., Cornier M.-A., White M.H., Hild A.K., Simonsen S.E., Melanson E.L., Halliday T.M. (2021). The Effects of Acute Exercise on Appetite and Energy Intake in Men and Women. Physiol. Behav..

[64] Middelbeek R.J.W., Motiani P., Brandt N., Nigro P., Zheng J., Virtanen K.A., Kalliokoski K.K., Hannukainen J.C., Goodyear L.J. (2021). Exercise Intensity Regulates Cytokine and Klotho Responses in Men. Nutr. Diabetes.

[65] Shakiba E., Sheikholeslami-Vatani D., Rostamzadeh N., Karim H. (2019). The Type of Training Program Affects Appetite-Regulating Hormones and Body Weight in Overweight Sedentary Men. Appl. Physiol. Nutr. Metab..

[66] Quist J.S., Rosenkilde M., Gram A.S., Blond M.B., Holm-Petersen D., Hjorth M.F., Stallknecht B., Sjödin A. (2019). Effects of Exercise Domain and Intensity on Sleep in Women and Men with Overweight and Obesity. J. Obes..

[67] Tremblay A., Dutheil F., Drapeau V., Metz L., Lesour B., Chapier R., Pereira B., Verney J., Baker J.S., Vinet A. (2019). Long-Term Effects of High-Intensity Resistance and Endurance Exercise on Plasma Leptin and Ghrelin in Overweight Individuals: The RESOLVE Study. Appl. Physiol. Nutr. Metab..

[68] Liao J., Huang J., Wang S., Xiang M., Wang D., Deng H., Yin H., Xu F., Hu M. (2021). Effects of Exercise and Diet Intervention on Appetite-Regulating Hormones Associated with miRNAs in Obese Children. Eat. Weight Disord. Stud. Anorex. Bulim. Obes..

[69] Dorling J.L., Clayton D.J., Jones J., Carter W.G., Thackray A.E., King J.A., Pucci A., Batterham R.L., Stensel D.J. (2019). A Randomized Crossover Trial Assessing the Effects of Acute Exercise on Appetite, Circulating Ghrelin Concentrations, and Butyrylcholinesterase Activity in Normal-Weight Males with Variants of the Obesity-Linked FTO Rs9939609 Polymorphism. Am. J. Clin. Nutr..

[70] Caldeira R.S., Panissa V.L.G., Inoue D.S., Campos E.Z., Monteiro P.A., de Melo Giglio B., Pimentel G.D., Hofmann P., Lira F.S. (2018). Impact to Short-Term High Intensity Intermittent Training on Different Storages of Body Fat, Leptin and Soluble Leptin Receptor Levels in Physically Active Non-Obese Men: A Pilot Investigation. Clin. Nutr. ESPEN.

[71] Inoue D.S., Panissa V.L., Antunes B.M., Oliveira F.P., Malta R.B., Caldeira R.S., Campos E.Z., Pimentel G.D., Franchini E., Lira F.S. (2018). Reduced Leptin Level Is Independent of Fat Mass Changes and Hunger Scores from High-Intensity Intermittent plus Strength Training. J. Sports Med. Phys. Fitness.

[72] Jackson M., Fatahi F., Alabduljader K., Jelleyman C., Moore J.P., Kubis H.-P. (2018). Exercise Training and Weight Loss, Not Always a Happy Marriage: Single Blind Exercise Trials in Females with Diverse BMI. Appl. Physiol. Nutr. Metab..

[73] Vardar S.A., Karaca A., Güldiken S., Palabıyık O., Süt N., Demir A.M. (2018). High-Intensity Interval Training Acutely Alters Plasma Adipokine Levels in Young Overweight/Obese Women. Arch. Physiol. Biochem..

[74] Martins C., Kulseng B., King N.A., Holst J.J., Blundell J.E. (2010). The Effects of Exercise-Induced Weight Loss on Appetite-Related Peptides and Motivation to Eat. J. Clin. Endocrinol. Metab..

[75] Bjersing L.J., Larsson A., Palstam A., Ernberg M., Bileviciute-Ljungar I., Löfgren M., Gerdle B., Kosek E., Mannerkorpi K. (2017). Benefits of Resistance Exercise in Lean Women with Fibromyalgia: Involvement of IGF-1 and Leptin|BMC Musculoskeletal Disorders. BMC Musculoskelet. Disord..

[76] Racil G., Zouhal H., Elmontassar W., Ben Abderrahmane A., De Sousa M.V., Chamari K., Amri M., Coquart J.B. (2016). Plyometric Exercise Combined with High-Intensity Interval Training Improves Metabolic Abnormalities in Young Obese Females More so than Interval Training Alone. Appl. Physiol. Nutr. Metab..

[77] Tan S., Wang J., Cao L., Guo Z., Wang Y. (2016). Positive Effect of Exercise Training at Maximal Fat Oxidation Intensity on Body Composition and Lipid Metabolism in Overweight Middle-Aged Women. Clin. Physiol. Funct. Imaging.

[78] Douglas J.A., King J.A., McFarlane E., Baker L., Bradley C., Crouch N., Hill D., Stensel D.J. (2015). Appetite, Appetite Hormone and Energy Intake Responses to Two Consecutive Days of Aerobic Exercise in Healthy Young Men. Appetite.

[79] Salvadori A., Fanari P., Brunani A., Marzullo P., Codecasa F., Tovaglieri I., Cornacchia M., Palmulli P., Longhini E. (2015). Leptin Level Lowers in Proportion to the Amount of Aerobic Work after Four Weeks of Training in Obesity. Horm. Metab. Res..

[80] Morishima T., Kurihara T., Hamaoka T., Goto K. (2014). Whole Body, Regional Fat Accumulation, and Appetite-Related Hormonal Response after Hypoxic Training. Clin. Physiol. Funct. Imaging.

[81] Dâmaso A.R., da Silveira Campos R.M., Caranti D.A., de Piano A., Fisberg M., Foschini D., de Lima Sanches P., Tock L., Lederman H.M., Tufik S. (2014). Aerobic plus Resistance Training Was More Effective in Improving the Visceral Adiposity, Metabolic Profile and Inflammatory Markers than Aerobic Training in Obese Adolescents. J. Sports Sci..

[82] Gholipour M., Kordi M.R., Taghikhani M., Ravasi A.A., Gaeini A.A., Tabrizi A. (2014). Possible Role for Growth Hormone in Suppressing Acylated Ghrelin and Hunger Ratings During and After Intermittent Exercise of Different Intensities in Obese Individuals. Acta Med. Iran..

[83] Sim A.Y., Wallman K.E., Fairchild T.J., Guelfi K.J. (2014). High-Intensity Intermittent Exercise Attenuates Ad-Libitum Energy Intake. Int. J. Obes..

[84] Zaccaria M., Ermolao A., Brugin E., Bergamin M. (2013). Plasma Leptin and Energy Expenditure during Prolonged, Moderate Intensity, Treadmill Exercise. J. Endocrinol. Investig..

[85] Heden T.D., Liu Y., Kearney M.L., Park Y., Dellsperger K.C., Thomas T.R., Kanaley J.A. (2013). Prior Exercise and Postprandial Incretin Responses in Lean and Obese Individuals. Med. Sci. Sports Exerc..

[86] Mendham A.E., Duffield R., Marino F., Coutts A.J. (2014). Small-Sided Games Training Reduces CRP, IL-6 and Leptin in Sedentary, Middle-Aged Men. Eur. J. Appl. Physiol..

[87] Tiryaki-Sonmez G., Ozen S., Bugdayci G., Karli U., Ozen G., Cogalgil S., Schoenfeld B., Sozbir K., Aydin K. (2013). Effect of Exercise on Appetite-Regulating Hormones in Overweight Women. Biol. Sport.

[88] Karacabey K. (2009). The effect of exercise on leptin, insulin, cortisol and lipid profiles in obese children. J. Int. Med. Res..

[89] Ahmadizad S., Ghorbani S., Ghasemikaram M., Bahmanzadeh M. (2014). Effects of Short-Term Nonperiodized, Linear Periodized and Daily Undulating Periodized Resistance Training on Plasma Adiponectin, Leptin and Insulin Resistance. Clin. Biochem..

[90] Loria-Kohen V., Fernández-Fernández C., Bermejo L.M., Morencos E., Romero-Moraleda B., Gómez-Candela C. (2013). Effect of Different Exercise Modalities plus a Hypocaloric Diet on Inflammation Markers in Overweight Patients: A Randomised Trial. Clin. Nutr. Edinb. Scotl..

[91] Thomas G.A., Kraemer W.J., Comstock B.A., Dunn-Lewis C., Volek J.S., Denegar C.R., Maresh C.M. (2012). Effects of Resistance Exercise and Obesity Level on Ghrelin and Cortisol in Men. Metabolism..

[92] Kraemer R.R., Francois M.R., Sehgal K., Sirikul B., Valverde R.A., Castracane V.D. (2011). Amylin and Selective Glucoregulatory Peptide Alterations during Prolonged Exercise. Med. Sci. Sports Exerc..

[93] Sartor F., de Morree H.M., Matschke V., Marcora S.M., Milousis A., Thom J.M., Kubis H.-P. (2010). High-Intensity Exercise and Carbohydrate-Reduced Energy-Restricted Diet in Obese Individuals. Eur. J. Appl. Physiol..

[94] Kosydar-Piechna M., Bilińska M., Janas J., Piotrowicz R. (2010). Influence of Exercise Training on Leptin Levels in Patients with Stable Coronary Artery Disease: A Pilot Study. Cardiol. J..

[95] Hagobian T.A., Sharoff C.G., Stephens B.R., Wade G.N., Silva J.E., Chipkin S.R., Braun B. (2009). Effects of Exercise on Energy-Regulating Hormones and Appetite in Men and Women. Am. J. Physiol. Regul. Integr. Comp. Physiol..

[96] King J.A., Miyashita M., Wasse L.K., Stensel D.J. (2010). Influence of Prolonged Treadmill Running on Appetite, Energy Intake and Circulating Concentrations of Acylated Ghrelin. Appetite.

[97] Hazell T.J., Islam H., Townsend L.K., Schmale M.S., Copeland J.L. (2016). Effects of Exercise Intensity on Plasma Concentrations of Appetite-Regulating Hormones: Potential Mechanisms. Appetite.

[98] Ozcelik O., Ozkan Y., Karatas F., Kelestimur H. (2005). Exercise Training as an Adjunct to Orlistat Therapy Reduces Oxidative Stress in Obese Subjects. Tohoku J. Exp. Med..

[99] Unick J.L., Otto A.D., Goodpaster B.H., Helsel D.L., Pellegrini C.A., Jakicic J.M. (2010). Acute Effect of Walking on Energy Intake in Overweight/Obese Women. Appetite.

[100] Crabtree D.R., Blannin A.K. (2015). Effects of Exercise in the Cold on Ghrelin, PYY, and Food Intake in Overweight Adults. Med. Sci. Sports Exerc..

[101] Larsen P.S., Donges C.E., Guelfi K.J., Smith G.C., Adams D.R., Duffield R. (2017). Effects of Aerobic, Strength or Combined Exercise on Perceived Appetite and Appetite-Related Hormones in Inactive Middle-Aged Men. Int. J. Sport Nutr. Exerc. Metab..

[102] Heden T.D., Liu Y., Park Y., Dellsperger K.C., Kanaley J.A. (2013). Acute Aerobic Exercise Differentially Alters Acylated Ghrelin and Perceived Fullness in Normal-Weight and Obese Individuals. J. Appl. Physiol..

[103] Guelfi K.J., Donges C.E., Duffield R. (2013). Beneficial Effects of 12 Weeks of Aerobic Compared with Resistance Exercise Training on Perceived Appetite in Previously Sedentary Overweight and Obese Men. Metabolism.

[104] Markofski M.M., Carrillo A.E., Timmerman K.L., Jennings K., Coen P.M., Pence B.D., Flynn M.G. (2014). Exercise Training Modifies Ghrelin and Adiponectin Concentrations and Is Related to Inflammation in Older Adults. J. Gerontol. A.

[105] Bowyer K.P., Carson J.A., Davis J.M., Wang X. (2019). The Influence of Exercise Training Dose on Fasting Acylated Ghrelin Concentration in Older Women. J. Behav. Med..

[106] Guadalupe-Grau A., Perez-Gomez J., Olmedillas H., Chavarren J., Dorado C., Santana A., Serrano-Sanchez J.A., Calbet J.A.L. (2009). Strength Training Combined with Plyometric Jumps in Adults: Sex Differences in Fat-Bone Axis Adaptations. J. Appl. Physiol..

[107] Leidy H.J., Gardner J.K., Frye B.R., Snook M.L., Schuchert M.K., Richard E.L., Williams N.I. (2004). Circulating Ghrelin Is Sensitive to Changes in Body Weight during a Diet and Exercise Program in Normal-Weight Young Women. J. Clin. Endocrinol. Metab..

[108] Chabot F., Caron A., Laplante M., St-Pierre D.H. (2014). Interrelationships between Ghrelin, Insulin and Glucose Homeostasis: Physiological Relevance. World J. Diabetes.

[109] Gao S., Moran T.H., Lopaschuk G.D., Butler A.A. (2013). Hypothalamic Malonyl-CoA and the Control of Food Intake. Physiol. Behav..

[110] Folmes C.D.L., Lopaschuk G.D. (2007). Role of Malonyl-CoA in Heart Disease and the Hypothalamic Control of Obesity. Cardiovasc. Res..

[111] Wolfgang M.J., Cha S.H., Sidhaye A., Chohnan S., Cline G., Shulman G.I., Lane M.D. (2007). Regulation of Hypothalamic Malonyl-CoA by Central Glucose and Leptin. Proc. Natl. Acad. Sci. USA.

[112] Hukshorn C.J., Saris W.H., Westerterp-Plantenga M.S., Farid A.R., Smith F.J., Campfield L.A. (2000). Weekly Subcutaneous Pegylated Recombinant Native Human Leptin (PEG-OB) Administration in Obese Men. J. Clin. Endocrinol. Metab..

[113] Cooper J., Watras A., Paton C., Wegner F., Adams A., Schoeller D. (2011). Impact of Exercise and Dietary Fatty Acid Composition from a High-Fat Diet on Markers of Hunger and Satiety. Appetite.

[114] Roth J.D., Roland B.L., Cole R.L., Trevaskis J.L., Weyer C., Koda J.E., Anderson C.M., Parkes D.G., Baron A.D. (2008). Leptin Responsiveness Restored by Amylin Agonism in Diet-Induced Obesity: Evidence from Nonclinical and Clinical Studies. Proc. Natl. Acad. Sci. USA.

[115] Zelissen P.M.J., Stenlof K., Lean M.E.J., Fogteloo J., Keulen E.T.P., Wilding J., Finer N., Rössner S., Lawrence E., Fletcher C. (2005). Effect of Three Treatment Schedules of Recombinant Methionyl Human Leptin on Body Weight in Obese Adults: A Randomized, Placebo-Controlled Trial. Diabetes Obes. Metab..

[116] Westerterp-Plantenga M.S., Saris W.H., Hukshorn C.J., Campfield L.A. (2001). Effects of Weekly Administration of Pegylated Recombinant Human OB Protein on Appetite Profile and Energy Metabolism in Obese Men. Am. J. Clin. Nutr..

[117] Mittendorfer B., Horowitz J.F., DePaoli A.M., McCamish M.A., Patterson B.W., Klein S. (2011). Recombinant Human Leptin Treatment Does Not Improve Insulin Action in Obese Subjects with Type 2 Diabetes. Diabetes.

[118] Moon H.S., Matarese G., Brennan A.M., Chamberland J.P., Liu X., Fiorenza C.G., Mylvaganam G.H., Abanni L., Carbone F., Williams C.J. (2011). Efficacy of metreleptin in obese patients with type 2 diabetes: Cellular and molecular pathways underlying leptin tolerance. Diabetes.

[119] Korner J., Conroy R., Febres G., McMahon D.J., Conwell I., Karmally W., Aronne L.J. (2013). Randomized Double-Blind Placebo-Controlled Study of Leptin Administration After Gastric Bypass. Obesity.

[120] Otelea M.R., Nartea R., Popescu F.G., Covaleov A., Mitoiu B.I., Nica A.S. (2022). The Pathological Links between Adiposity and the Carpal Tunnel Syndrome. Curr. Issues Mol. Biol..

[121] Nartea R., Mitoiu B.I., Ghiorghiu I. (2023). The Link between Magnesium Supplements and Statin Medication in Dyslipidemic Patients. Curr. Issues Mol. Biol..

[122] Jin X., Qiu T., Li L., Yu R., Chen X., Li C., Proud C.G., Jiang T. (2023). Pathophysiology of Obesity and Its Associated Diseases. Acta Pharm. Sin. B.

[123] Friedman J.M. (2019). Leptin and the Endocrine Control of Energy Balance. Nat. Metab..

[124] Zhao S., Zhu Y., Schultz R.D., Li N., He Z., Zhang Z., Caron A., Zhu Q., Sun K., Xiong W. (2019). Partial Leptin Reduction as an Insulin Sensitization and Weight Loss Strategy. Cell Metab..

[125] Heymsfield S.B., Greenberg A.S., Fujioka K., Dixon R.M., Kushner R., Hunt T., Lubina J.A., Patane J., Self B., Hunt P. (1999). Recombinant Leptin for Weight Loss in Obese and Lean Adults: A Randomized, Controlled, Dose-Escalation Trial. JAMA.

