# Supplementary material for: Impact of Resistance and Endurance Training on Ghrelin and Plasma Leptin Levels in Overweight and Obese Subjects
Source: Int J Mol Sci. 2024 Jul 24;25(15):8067. doi: 10.3390/ijms25158067 (PMC11311634; doi:10.3390/ijms25158067)
Supplement: Supplementary file 1 [file ijms-25-08067-s001.zip › ijms-3047358-supplementary.pdf]

Supplement Table S1

Study (reference/year)	Participants (number/ characteristics/years)	Exercise type/duration/intensity	Ghrelin form, leptin, method	Results
**Adults**				
Heinen D et al 2023 [87]	105 adults with major depression	Acute bicycle ergometer test and Aerobic training with endurance exercise for 12 weeks	Leptin, EIA	-no significant differences between depressed patients and control<br>- significant relations between leptin level and body weight and body fat content<br>-no significant changes in circulating leptin levels between acute and chronic training
Li S, Guo R et al. 2023 [69]	14 obese adults	1) M group (MICE without BFR, 60%VO2max, 200 kJ); (2) B group (MICE with BFR, 60%VO2max, 200 kJ); and (3) C group (control session without exercise).	total ghrelin, RIA	combined MICE and BFR exercise reduced the appetite of obese adults by promoting the secretion of Lac-Phe and ghrelin
Beer NJ et al. 2022 [78]	36 inactive men and women (body mass index 29.6±3.8kg/m²)	Three sessions per week of SIT (alternating cycling for 15 s at 170% V˙O2peak and 60 s at 32% V˙O2peak) with need-support or traditional moderate-intensity continuous training (MICT) without need-support (continuous cycling at 60% V˙O2peak).	Active ghrelin, leptin, RIA	Post-exercise energy intake from snacks decreased significantly from pre- (807 ± 550 kJ) to post-SIT (422 ± 468 kJ; p < 0.05) but remained unaltered following MICT.
Halliday et al.,2021 [29]	24 adults 35%+/-2% body fat	AEx (Walking at 65%-70% heart rate max for 45 minutes)<br>Rex(1 set to failure of 12 exercises)<br>SED (sedentary control group)	Total ghrelin, EIA	- ghrelin, PYY, and GLP-1 were all lower after REx versus AEx (ghrelin: 130,737 ± 4928 for REx; 143,708 ± 7500 for AEx (P = 0.006); PYY: 20,540 ± 1177 for REx, 23,812 ± 1592 for AEx (P = 0.001); and GLP-1: 1314 ± 93 for REx, 1615 ± 110 for AEx (P = 0.013)).
Tobin SY et al.,2021/ [62]	24 Men and women (n = 24; 50% male) with overweight/obesity, matched on age (32.3 ± 2 vs. 36.8 ± 2 yrs, p = 0.14) and BMI (28.1 ± 1.2 vs 29.0 ± 1.5 kg/m2, p = 0.64)	1) AEx (65-70% of age-predicted maximum heart rate for 45 min);<br>2) REx (1-set to failure on 12 exercises);<br>3) CON.	TOTAL GHRELIN AND PYY RIA	There were no statistically significant differences between men and women, nor were there any sex-by-condition differences in these appetite-related hormones (all. P> 0.05).<br>Acute REx lowers both orexigenic (ghrelin) and anorectic (PYY and GLP-1) gut peptides compared to acute AEx. Ad libitum energy intake did not increase compared to SED in either exercise condition, indicating both exercise modalities have appetite and energy intake-suppressing effects.
Fico BG et al. 2020 [54]	32 adults with obesity and osteoarthritis	Twelve weeks of supervised swimming or cycling training. In the initial few weeks, participants exercised for 20–30 minutes/day, three days/week, at an exercise intensity of 40–50% of heart rate reserve (HRR). Subsequently, the intensity and duration of exercise were progressively increased to 40–45 minutes/day, three days/week, at an intensity of 60–70% of HRR.	Leptin, acylated ghrelin using RIA	Leptin and active ghrelin concentrations did not change with long-term exercise training.

Heiston et al. 2019 [28]	28 age: 61.3 ± 1.5 yr; body mass index (BMI): 33.2 ± 1.1 kg/m2] with prediabetes	short-term moderate-continuous (CONT) vs. high-intensity interval (INT) training on appetite regulation.	Acylated ghrelin (AG), des-acylated ghrelin (DAG), using RIA	Two weeks of exercise, independent of intensity, does not alter postprandial appetite hormones or hunger despite slight reductions in food intake and weight.
Quist JS et al. 2019 [80]	130 physically inactive women and men (20-45 yr) with overweight and obesity were	randomized to 6 mo of habitual lifestyle (CON, n = 18), active commuting (BIKE, n = 35), or leisure-time exercise of moderate [MOD, 50% peak oxygen uptake (Vo2peak)-reserve, n = 39] or vigorous (VIG, 70% Vo2peak-reserve, n = 38) intensity.	Acylated ghrelin, RIA	At three mo, basal appetite ratings and hormone concentrations did not change in any of the intervention groups compared with CON ($P \geq 0.11$ for all comparisons), except for glucagon, which was lower in BIKE [−32 (−63; −1)%, $P < 0.05$] and VIG [−38 (−68; −8)%, $P = 0.01$] compared with CON<br>At six mo, basal GLP-1 was higher in VIG compared with CON [25 (1; 47)%, $P = 0.04$], and basal ratings of hunger were higher in BIKE compared with VIG ($P = 0.03$), whereas basal acylated ghrelin was higher in VIG than in BIKE ($P = 0.02$) and MOD ($P = 0.01$)
Tremblay A, et al. 2019 [86]	100 inactive overweight adults/elderly with MetS (56 females, 44 males) (50–70)	Three exercise interventions: high-resistance-low-aerobic exercise (Re), low-resistance-high-aerobic exercise (rE), and low-resistance-low-aerobic exercise (re).	Acylated Ghrelin, RIA, leptin	Ghrelin was significantly increased after day 21 and month 3 ($p < 0.001$) and returned to a level comparable to baseline between months 6 and 12 when body weight and fat had reached a plateau.<br>Leptin levels decreased during the first three months and then plateaued.
Yu AP et al. l.2018 [53]	79 centrally obese MetS subjects aged 58± eight years<br>39 subjects received one year of yoga training<br>40 subjects received no training	One year of yoga training	Ghrelin, Acylated ghrelin (AG), EIA	Yoga training significantly increased circulating GH (control: -3%; yoga: +22%), total circulating ghrelin (control: -26%; yoga: +13%), and UnAG (control: -27%; yoga: +14%)
Martins C et al. 2015 [27]	12 overweight/obese volunteers	acute isocaloric bouts (250 kcal) of high-intensity intermittent cycling (HIIC) and moderate-intensity continuous cycling (MICC) or short-duration HIIC (S-HIIC) (125 kcal) and a resting control condition	Acylated Ghrelin, RIA	Acylated ghrelin plasma levels were lower in the MICC and HIIC but not in S-HIIC, compared with those in control.
Salvadori A. et al 2015 [104]	Eight subjects	Group A: aerobic training schedule<br>Group B: aerobic training program with a about of work beyond the anaerobic threshold (AT)	Leptin, RIA	reduction in leptin levels in both groups<br>Aerobic training alone appears to be linked to a more significant leptin reduction.
Morishima, T. et al. 2014 [102]	20 sedentary subjects	4-week training at 55% of maximal oxygen uptake (V·O(2max)) for hypoxic and normoxic – 3 times/week	Ghrelin, EIA<br>Leptin, RIA	-A significant reduction of postprandial leptin response was observed in both groups<br>-postprandial plasma ghrelin was similar in both groups
Loria-Kohen V et all 2012 [94]	119 overweight subjects	Four groups – strength training, endurance training, combined training, diet+physical recommendations	Leptin	-leptin decreased significantly in all groups
**Males**				
Rostamzadeh, N et al 2022 [70]	30 obese males	two groups of 15 individuals, including the RT group (3 sessions per week, four sets of 8 repetitions with 80% of 1RM) and the control group.	Acylated Ghrelin, RIA	plasma concentrations of acylated ghrelin and insulin hormones, as well as body weight, BMI, WHR, and body fat percentage, were significantly lower in the experimental group compared to the control group after 2, 4, and 6 months of RT (in all cases, $p \leq 0.05$). However, after 4 and 6 months of RT, the levels of peptide YY were higher in the training group compared to the control group ($p = 0.001$ and $p = 0.001$,

				respectively). Within-group changes indicated acylated ghrelin reduced (mean difference, −9.27 pg/dl; P = 0.001), while PYY increased (mean difference, 2.66 pg/dl; P = 0.001) after six months of RT compared to the pre-test.
Middelbeek RJW et al. 2021 [88]	22 healthy sedentary males	Sprint intensity training (SIT) consisted of six sessions over two weeks of × six 30-second all-out cycle ergometer sprints with four minutes of recovery between sprints.<br>-moderate-intensity training (MIT) consisted of 6 sessions over two weeks of cycle ergometer exercise at 60% VO_{2peak}, gradually increasing in duration from 40 to 60 min.	Leptin, RIA	Both training programs reduced the concentrations of plasma leptin.
Ouerghi et al. 2019 [8]	Seven inactive, overweight middle-aged males (36.4 ± 4.35)	Moderate exercise: 60% of PAP, 20 min<br>Heavy exercise: 80% of PAP, 20 min	Ghrelin, RIA	There is no significant difference in ghrelin level
Shakiba E et all. 2019 [81]	44 overweight men<br>Four groups of 11 individuals, which included (i) endurance group (3 sets of 10 min with 80%-90% of maximum heart rate), (ii) resistance group (4 sets of 8 repetitions with 80% of 1-repetition maximum), (iii) concurrent group (combination of programs of endurance and resistance groups in an alternate manner), and (iv) control group.	12 weeks for three sessions per week	Acylated Ghrelin, RIA	decreased serum acylated ghrelin (p = 0.000, p = 0.000, and p = 0.004<br>a significant positive correlation between weight (p = 0.003) and BMI (p = 0.009) changes with ghrelin
Murawska-Cialowicz E. et al. 2022 [85]	75 females	Nine weeks of training, assessment after one session, 3m, 6m, 9m	Leptin, RIA	Leptin was significantly decreased in all measurements
Dorling JL et al.2019 [79]	24 males with A or T allele for the obesity-linked FTO rs9939609 polymorphism (21 ± 3.55)	Running exercise, 70% of VO2max, 60 min	Acylated Ghrelin, RIA	Exercise increases BChE activity, suppresses AG and the AG: DAG ratio, and corrects the higher AG profile observed in obesity-risk AA individuals
Caldeira RS et al. 2018 [89]	20 men	Five weeks of HIIT or five weeks of SST- 3 times/week.	Leptin, RIA	Leptin was reduced in both groups.<br>There was no effect on body composition and hunger perception.
Inoue D et al. 2018 [96]	16 men	combined HIIT followed by strength exercise, two times/week for eight weeks,	Leptin, EIA	Leptin was reduced in combined HIIT plus strength training independent of body composition and hunger index alterations.
Holliday A. et al. 2017 [30]	Eight overweight individuals	4 × 30 s "flat-out" cycling on an ergometer (adapted Wingate test). Two hours post-exercise (or REST),	Acylated Ghrelin, RIA	Acylated ghrelin concentration was suppressed in EX compared with REST immediately post-exercise (113.4 ± 43.0 pg·mL-1 vs. 189.2 ± 91.8 pg·mL-1, p = 0.03, d = 1.07) and remained lower until the ad libitum test-meal
Martins C et al. 2017 [71]	45 sedentary obese individuals (30 women and 16men with BMI 33.3± 2.9 kg/m2)	the effect of 12 weeks of isocaloric programs of moderate-intensity continuous training (MICT), high-	Acylated Ghrelin, RIA	No significant effect of exercise intervention was found on the plasma concentration of acylated ghrelin.

		intensity interval training (HIIT), or short-duration HIIT		
Gibbons et al. 2017 [56]	32 overweight/obese individuals	16 completed 12 weeks of aerobic exercise program<br>16 Non EX	Acylated Ghrelin, RIA	Weight losers showed greater suppression of acylated ghrelin and more significant release of GLP-1 and PYY at baseline.
Gholipour M et al. 2014 [75]	Nine inactive males	ran on the treadmill at 0900 with progressive intensities of 50, 60, 70, and 80% of VO2max for 10, 10, 5, and 2 min respectively	Des-Acylated ghrelin (DAG), RIA	acylated ghrelin concentrations and hunger ratings are suppressed during exercise and two hours after
Sim AY, 2014 [77]	17 overweight men (body mass index: 27.7±1.6 kg m(-2)	CON: resting control,<br>MC: continuous moderate-intensity exercise (60% VO(2peak)),for 30 minutes<br>HI: high-intensity intermittent exercise (alternating 60 s at 100% VO(2peak) and 240 s at 50% VO(2peak)), for 30 minutes<br>VHI: very-high-intensity intermittent exercise (alternating 15 s at 170% VO(2peak) and 60 s at 32% VO(2peak)) for 30 minutes.	Active ghrelin EIA	lower active ghrelin (P≤0.050) after VHI compared with all other trials<br>There was an interaction effect of trial and time on the circulating levels of active ghrelin (P¼ 0.001), with lower active ghrelin levels immediately post VHI compared with CON, MC, and HI (Pp0.050). There was also a main effect of time on active ghrelin (P¼ 0.001), with a lower level at 35 min (P¼ 0.007) and 65 min post-exercise (P¼ 0.007) compared with the baseline.
Douglas JA et al.2015 [57]	15 healthy males	Treadmill exercise, 60% of VO2peak, 60 min – 2 days	Acylated Ghrelin, EIA<br>Leptin, EIA	high volume exercise does not stimulate changes in leptin and acylated ghrelin
Mendham A. et al. 2014 [103]	33 middle-aged, sedentary men	Three days/week for eight weeks	Leptin, EIA	Leptin levels significantly decreased
Zaccaria M, et three-time[93]	Seven males	4-h treadmill exercise. The starting intensity was set at 65% of maximal oxygen consumption – only one session	Leptin, EIA	Leptin levels significantly decreased
Ahmadizad S et al. 2013 [106]	Thirty-two sedentary overweight men	RT protocols three days/week for eight weeks	Leptin, EIA	-no significant changes were observed in plasma adiponectin and leptin concentrations between groups<br>-Body fat percent and waist-to-hip ratio (WHR) decreased significantly<br>-No changes in BMI
Thomas GA et al.2011 [63]	19 obese males	Resistance exercises (6 exercises, 3 sets of 10 repetitions at 85-95%, 10 repetitions at maximum with 120-90 seconds rest)	Total Ghrelin, RIA	only WHO 2/3 of obese men had significantly greater concentrations of ghrelin when compared with lean and WHO 1 obese group
Kraemer R et al. 2011 [91]	80 young males	standard nutrient beverage, after 1,5h, subjects initiated 90 min of treadmill exercise at 60% of $\dot{V}O_{2max}$.	Leptin, RIA	Exercise increased leptin concentrations after 5 min at 90% of VO2 max, then decreased to resting values during recovery.
Kosydar-Piechna M. et al. 2010 [90]	64 males with stable coronary artery disease	-6 weeks of aerobic training, three times/week, at 60-80% of maximal heart rate	Leptin, RIA	Leptin levels did not change in the study group<br>Leptin levels increased in the control group
Hagobian TA et al. 2009 [66]	Nine overweight and young adult males, VO2peak, 44.9 ± 4.8 (26.8 ± 11.8)	Treadmill running, 50–65% of VO2peak until 30% of total daily energy expenditure, 83 ± 8 min	Acylated Ghrelin, RIA	In men, the acylated ghrelin area under the curve (AUC) was not different between conditions. In women, acylated ghrelin AUC was higher after DEF (+32%) and BAL (+25%), and the change from baseline was higher than in men ($P < 0.05$).

Martins C et al. 2010 [71]	22 sedentary overweight/obese individuals (age 36.9± 8.3years;BMI 31.3± 3.3kg/m²)	a 12-week supervised exercise program (five times per week, 75% maximal heart rate), and they were requested not to change their food intake during the study.	Total Ghrelin, Acylated Ghrelin (AG), EIA	A significant increase (127%) in the suppression of AG postprandially<br>Exercise resulted in a substantial reduction in body weight and fasting insulin and an increase in AG plasma levels and fasting hunger sensations
Sartor F et al. 2010 [92]	19 obese adults	CHO-reduced diet+energy-restricted diet – 2 weeks<br>Diet+HIIT (10 times)	Leptin, RIA	Plasma leptin was reduced in both groups
**Females**				
Oh DH, Lee JK et al.2023 [84]	16 females >40yr	resistance and either moderate (RME, 50% VO_2max, 200 kcal [*n* = 8]) or vigorous aerobic exercise groups (RVE, 80% VO_2max, 200 kcal [*n* = 8]), respectively. After eight weeks of exercise,	Leptin, RIA	leptin levels decreased significantly in both groups
Murawska-Cialowicz E. et al. 2022 [85]	75 females	Nine weeks of training, assessment after one session, 3m, 6m, 9m	Leptin, RIA	Leptin was significantly decreased in all measurements
Kang SJ et al. 2018 [55]	13 middle-aged obese females	Aerobic + resistance exercise training, 50 min, five times/wk, 12 wks	Ghrelin, EIA<br>Leptin, EIA	Leptin was significantly decreased, and ghrelin was increased considerably.
Jackson M et al. 2018 [100]	70 overweight/obese (OV/OB) and lean female<br>34 females finished the 4-week intervention, and 36 females the 8-week intervention<br>Two single-blind exercise trials (4 weeks (study 1) and eight weeks (study 2)	Three times a week (45-90 min), intensity 50%-90% peak oxygen uptake for 4 and 8 weeks	Ghrelin, EIA<br>Leptin, RIA	Appetite hormone levels were significantly ($p < 0.05$) altered in the OV/OB group, affecting fasting (-24%) and postprandial amylin (-14%) levels. Investigating individuals' BMI responses using multiple regression analysis revealed that fasting leptin, postprandial amylin increase, and BMI were significant predictors of BMI change, explaining about 43% of the variance.
Bjersing JL et al. 2017 [98]	43 females with fibromialgia	progressive resistance exercise, twice weekly for 15 weeks	Leptin, EIA	Leptin was significantly reduced in lean women
Racil G et al. 2016 [105]	68 young obese females	-12 weeks of high-intensity interval training (HIIT) with the<br>-12 weeks of plyometric exercise combined with HIIT (P+HIIT)	Leptin, EIA	The P+HIIT program induced greater improvements in leptin concentrations.
Tan S. et al. 2016 [97]	30 middle-aged females	-5 days per week and 1 hour per day for ten weeks	Leptin, RIA	Leptin was significantly decreased
Mason C et al. 2015 [51]	439 overweight or obese postmenopausal women	A 12-month randomized controlled trial comparing: i) dietary weight loss with a 10% weight loss goal ('diet'; n=118); ii) moderate-to-vigorous intensity aerobic exercise for 45 min/day, five days/week ('exercise'; n=117); iii) dietary weight loss and exercise ('diet + exercise'; n=117); or iv) no-lifestyle-change control (n=87	Total Ghrelin, RIA	Fasting total ghrelin significantly increased in the diet + exercise arm (+7.4%, p=0.008) but not in either the diet (+6.5%, p=0.07) or exercise (+1.0%, p=0.53) arms compared to control. Despite the intervention, more significant weight loss was associated with increased ghrelin concentrations.
Heden TD et al. 2013 [72]	14 obese, mildly active females, VO2peak, 49 ± 7.3 mL·kg−1·min−1 (25.1 ± 5)	Treadmill walking, 55–60% of VO2peak, 60 min<br>NoExercise group	Acylated Ghrelin, RIA	no changes in fasting or postprandial acylated ghrelin concentrations with Exercise and postprandial fullness was attenuated by 46% compared with the NoEXercise group (P = 0.05).

Tiryaki-Sonmez G et al. 2013 [68]	Nine untrained overweight females (22.8 ± 1.38)	Treadmill exercise, 50% of VO2max, 60 min	Acylated Ghrelin, RIA	statistically insignificant (P > 0.05)
Guadalupe-Grau, A. et al. 2009 [95]	66 young sedentary adults	9-week strength combined with a plyometric jumps training program	Leptin, EIA	Leptin concentration negatively correlated to osteocalcin Leptin concentration is reduced with training in women
**Adolescents and children**				
Najafi R et al, 2023/[52]	30 girls /6-8 years - old/precocious puberty -15 girls -medication (GnRH) - 15 girls -medication (GnRH) + training	aerobic training program for three days/week with 20-75 min per day and 45-75% of maximum heart rate for 12 weeks.	Total ghrelin, EIA (enzyme immunoassay) Leptin, EIA	- leptin significantly decreased (p = 0.001) and ghrelin significantly increased (p = 0.001) in the medication+ training group - no significant difference was observed in the ghrelin (p = 1) and leptin (p= 0.78) in the medication group. Ghrelin levels were negatively correlated with leptin and BMI.
Liao J et al. 2019 [74]	16 obese children	exercise and diet intervention for six weeks	Total ghrelin, RIA	ghrelin was significantly enhanced (P < 0.05) after six weeks
Vardar S, et al.2018 [99]	12 overweight/obese young females	19 days of HIT comprising six sessions of 4-6 repeats of a Wingate test	Leptin, EIA	Plasma leptin concentrations decreased 5 min after exercise and remained reduced following 90 min in both the first and last training days.
Damaso AR. Et al. 2014 [101]	139 obese adolescents	Aerobic training (AT) + Resistance training (RT) - 1year	Leptin/adiponectin ratio	Improvement in the leptin/adiponectin ratio, especially for the patients in AT+RT
Campos RM et al. 2014 [32]	42 post-pubertal obese adolescents (28 females, 14 males)	Aerobic training, 60 min, three times/wk, 52 weeks Aerobic + resistance training, 2 × 30 min, 52 weeks	Ghrelin, EIA	Ghrelin level showed a significant increase at six months and nine months (P < 0.05), while leptin, insulin, and HOMA were significantly decreased at all three-time points Tested(3, 6, and 9 months)
Jones TE et al. 2012 [33]	12 overweight adolescents, ≥ the 85th percentile for BMI for age and sex	an 8-month supervised aerobic training program (5-minute warm-up of light stretching, 45 minutes of aerobic training at 60–85% of measured peak oxygen uptake (VO2), and 5–10 minutes of cool-down activity)	Active ghrelin, leptin, EIA	Leptin and active ghrelin concentrations did not change with long-term exercise training.
Gueugnon C et al. 2012 [31]	32 obese inactive adolescents (22 females, ten males) BMI z score = 4.1)- one year in an institution for childhood obesity a group of 15 normal-weight teenagers recruited from the community (5 boys and 10 girls) as a control group	physical exercise for 45 to 60 minutes at least five times per week	Total Ghrelin, RIA	increase in ghrelin (apparent from month 6; P < 0.05), a decrease in leptin (from month 3)

Abbreviations: AG-acylated ghrelin; AT aerobic training; BChE- Butyrylcholinesterase; BFR- blood flow restriction; BMI Body Mass Index; CONT short-term moderate continuous training; EIA enzyme immunoassay; FiO2 fractional inspired oxygen; Gn-RH gonadotropin-releasing hormone; HIIC high-intensity interval cycling; HIIT high-intensity interval training; HRmax maximum heart rate; HRR heart rate reserve; HRR heart rate reserve; INT high-intensity interval training; LIA luminescence immunoassay; MAP of maximal aerobic power; MetS metabolic syndrome; MICE- moderate-intensity Continous exercises; MICT moderate-intensity continuous training; MIIC moderate-intensity continuous cycling; OB obese; OV overweight; P+HIIT pleyotropic+ high-intensity interval training; Re high resistance low aerobic exercise; rE low resistance high aerobic exercise; re- low resistance, low aerobic exercise; RES resistance exercises; RES resistive; RIA radioimmunoassay; RM repetition maximum; RME resistance moderate exercise; RT resistive training; RVE vigorous aerobic exercise; S-HIIC short-duration high-intensity; SIT sprint intensity training; SST steady-state training; TV training volume; UnAG- unacylated ghrelin; VO2max maximal oxygen uptake; VO2peak peak of oxygen consumption; WHO World Health Organization.
